# An investigation of the impact of using contrast- and arm-synthesis models for network meta-analysis

**DOI:** 10.1017/rsm.2025.18

**Published:** 2025-04-25

**Authors:** Amalia Karahalios, Ian R. White, Simon L. Turner, Georgia Salanti, G. Peter Herbison, Areti Angeliki Veroniki, Adriani Nikolakopoulou, Joanne E. McKenzie

**Affiliations:** 1 Methods in Evidence Synthesis Unit, School of Public Health and Preventive Medicine, Monash University, Melbourne, VIC, Australia; 2 Centre for Epidemiology and Biostatistics, Melbourne School of Population and Global Health, University of Melbourne, Parkville, VIC, Australia; 3 MRC Clinical Trials Unit at UCL, University College London, London, UK; 4 Institute of Social and Preventive Medicine, University of Bern, Bern, Switzerland; 5 University of Otago, Dunedin, New Zealand; 6 Knowledge Translation Program, Li Ka Shing Knowledge Institute, St. Michael’s Hospital, Unity Health Toronto, Toronto, ON, Canada; 7 Institute for Health Policy, Management, and Evaluation, University of Toronto, Toronto, ON, Canada; 8 Laboratory of Hygiene, Social and Preventive Medicine and Medical Statistics, School of Medicine, Aristotle University of Thessaloniki, Thessaloniki, Greece; 9 Institute of Medical Biometry and Statistics, Faculty of Medicine and Medical Centre–University of Freiburg, Freiburg, Germany

**Keywords:** arm-based, contrast-based, empirical evaluation, indirect treatment comparison, mixed-treatment comparison, network meta-analysis

## Abstract

Network meta-analysis allows the synthesis of relative effects from several treatments. Two broad approaches are available to synthesize the data: arm-synthesis and contrast-synthesis, with several models that can be fitted within each. Limited evaluations comparing these approaches are available. We re-analyzed 118 networks of interventions with binary outcomes using three contrast-synthesis models (CSM; one fitted in a frequentist framework and two in a Bayesian framework) and two arm-synthesis models (ASM; both fitted in a Bayesian framework). We compared the estimated log odds ratios, their standard errors, ranking measures and the between-trial heterogeneity using the different models and investigated if differences in the results were modified by network characteristics. In general, we observed good agreement with respect to the odds ratios, their standard errors and the ranking metrics between the two Bayesian CSMs. However, differences were observed when comparing the frequentist CSM and the ASMs to each other and to the Bayesian CSMs. The network characteristics that we investigated, which represented the connectedness of the networks and rareness of events, were associated with the differences observed between models, but no single factor was associated with the differences across all of the metrics. In conclusion, we found that different models used to synthesize evidence in a network meta-analysis (NMA) can yield different estimates of odds ratios and standard errors that can impact the final ranking of the treatment options compared.

## Highlights

### What is known

Two broad approaches have been proposed to synthesize the evidence in a NMA, and there has been heated debate about which model should be used. The CSMs combine the relative treatment effects over the trials, whereas the ASMs combine the arm-level summaries, and relative treatment effects are then constructed. Previous research has established the conditions under which bias in the treatment effects could arise, but it is not clear in practice if these conditions are likely to arise.

### What is new

We analyzed data from 118 networks with binary outcomes using five synthesis models (three contrast-synthesis and two arm) and investigated if four characteristics of the networks explained the observed differences between the models. We found important differences in the estimates obtained from the CSMs and ASMs. Specifically, the different models can yield different estimates of odds ratios and standard errors, leading to differing surface under the cumulative ranking curve (SUCRA) values that can have an impact on the final ranking of the treatment options compared. Of the four factors that we investigated, no single factor was found to explain the differences across all of the metrics.

### Potential impact for RSM readers

NMA models are commonly used to synthesize evidence from multiple treatments. This paper highlights that it is imperative for authors to consider and pre-specify their NMA model.

## 1 Introduction

Network Meta-Analysis (NMA) is an extension to pairwise meta-analysis which allows the synthesis of relative effects from more than two treatments. To synthesize the evidence in an NMA, two broad approaches have been proposed.^1^ ^–^ ^4^ The validity of these approaches has been hotly debated, leading to polarization of opinions on which model should be used.^1^ ^,^ ^5^ Discussion of when the approaches are equivalent and their merits and disadvantages has continued in a collection of publications reflecting on twenty years of NMA.^6^ ^–^ ^9^ In the first approach, the relative treatment effects (e.g., natural log odds ratio 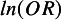 , or mean difference) over the trials are combined (henceforth referred to as contrast-synthesis model, CSM).^10^ ^,^ ^11^ In the second approach, the arm-level summaries (e.g., natural log odds, mean) are combined in a suitable statistical model, and relative treatment effects are then constructed (henceforth referred to as arm-synthesis model, ASM).^3^ ^,^ ^9^ ^,^ ^12^ The CSMs, which are the standard models used in meta-analysis, have intuitive appeal because they rely on only within-study information and therefore respect randomization.^1^ The ASMs have the advantage of being able to compute various estimands within the model (e.g., marginal risk difference).^3^ ^,^ ^9^ ^,^ ^12^ However, these models have been criticized because they compromise randomization and therefore have the potential to yield biased estimates of the relative treatment effects.^1^ The conditions under which bias in the treatment effects could arise have been established^13^; however, it is not clear in practice if these conditions are likely to arise and what the magnitude of bias would be.

When fitting the models, several decisions need to be made. For one, a decision needs to be made regarding whether the model will be fitted within a Bayesian or frequentist framework. For another, when fitting a CSM using random effects where a common between-trial heterogeneity variance of the relative treatment effects is assumed,^4^ ^,^ ^10^ ^,^ ^14^ a decision needs to be made as to whether the heterogeneity variance (i.e., between trial variance, 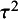 ) is estimated from information in the network or incorporates external information on heterogeneity (such as estimated heterogeneity from large empirical data sets).^15^

White et al.^13^ showed theoretically how the ASMs and CSMs differ, and using a small, hypothetical dataset, found that important differences between the models emerge dependent on the implementation of the models (i.e., when random study intercepts are fitted, see White et al.^13^ for more details). Three simulation studies have been undertaken that investigated different models for fitting NMAs under very specific scenarios.^3^ ^,^ ^16^ ^,^ ^17^ One study, published in 2024, compared the models using three hypothetical datasets.^18^ To the best of our knowledge, only one empirical evaluation has been undertaken to examine the impact of using ASMs and CSMs on the results.^19^ However, the evaluation was restricted to 14 networks and examined a limited number of models.

The aim of this study was to empirically compare the results of NMA between the CSMs and ASMs with several implementations of each model when applied to a large number of real-world networks of binary outcomes. Furthermore, we aimed to investigate whether characteristics of the networks were associated with the magnitude of differences in results between the methods. This empirical study will complement the work of White et al.^13^

## 2 Methods/Design

A protocol for this study has been published.^20^ An overview of the methods is provided here, with deviations from the planned methods presented in Supplementary Table S1.

### 2.1 Database of networks

We used a database of 456 NMAs of randomized trials that had been previously identified and curated.^21^ ^,^ ^22^ Further details on the methods for locating the NMA publications, inclusion criteria, screening process and the data extracted are available.^20^ ^,^ ^21^

### 2.2 Eligibility criteria for the present study

We included a subset of networks from the database which met the following inclusion criteria: i) the primary outcome of the network was binary, ii) the number of events and number of participants were available for each study-specific direct comparison within the network, iii) for networks including at least one loop, there was no evidence of inconsistency in the network as detected by a *p*-value >0.10 via the design-by-treatment interaction test using the **mvmeta** command in Stata,^23^ and iv) the network had a control/placebo arm. We focused on binary outcomes because the majority of NMAs are of this type.^24^ We included networks with no evidence of inconsistency because the models fitted do not allow for inconsistency.

### 2.3 Statistical models used to analyze the networks

We analyzed each NMA using three CSMs and two ASMs (collectively referred to throughout as ‘synthesis models’). The mathematical details of the synthesis models fitted are available in the protocol.^20^ A full description of each model, likelihood and the informative priors is provided in Table 1, and an overview of the details of the implementation of the synthesis models follows. Three CSMs were chosen to enable comparison of the CSM implemented in a Bayesian framework with a vague prior uniform distribution for the between-study variance, 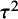 (CSM1), and an informative prior distribution (CSM2), and implemented in a frequentist framework (CSM3). The ASMs were selected to enable comparison of different variance assumptions of the random effects: homogeneity of variances of the random effects (ASM1), and heterogeneity of variances of the random effects (ASM2). Note that for both models (ASM1 and ASM2) the correlations between the random effects within studies are assumed equal. Under the assumption of homogeneity, the variance of the log(odds) is assumed to be the same across different treatments, whereas under the assumption of heterogeneity, the variances are assumed to differ. Importantly, the comparison of CSM1 with ASM1 provides the most direct comparison between contrast-synthesis and arm-synthesis approaches. This is because the CSMs and ASM1 assume that the variance is constant (i.e., independent of the treatment), whereas ASM2 allows the variance to depend on the treatments.^13^

**Table 1:** Overview of the methods applied to synthesize the evidence from network meta-analyses

							Prior distributions		
								Mean	
							Treatment	effect of	Heterogeneity
	Description		Contrast-level	Frequentist	Likelihood		specific	treatment k	or random
Method	of model and	Package	or arm-level	or Bayesian	and link		fixed	relative to	effects
label	likelihood	used in R	input data	framework	functions	Heterogeneity	effects	baseline	parameter
Contrast-synthesis model 1 (CSM1)	Contrast-based model, arm-based likelihood	gemtc (version 0.8.1)	Arm-level	Bayesian	Binomial likelihood and logit link	Common	N/A	δ_k_ ~ N(0, (15*5)^2^)^a^	τ_bk_ ~ U(0,10)^b^
Contrast-synthesis model 2 (CSM2)	Contrast-based model, arm-based likelihood	gemtc (version 0.8.1)	Arm-level	Bayesian	Binomial likelihood and logit link	Common	N/A	δ_k_ ~ N(0, (15*5)^2^)^a^	Informative^c^
Contrast-synthesis model 3 (CSM3)	Contrast-based model and likelihood	netmeta (version 0.9–2)	Contrast-level	Frequentist	N/A	Common	N/A	N/A	N/A
Arm-synthesis model 1^d^ (ASM1)	Arm-based model and likelihood	pcnetmeta (version 2.4)	Arm-level	Bayesian	Binomial likelihood and probit link	Common	μ_k_~ N(0, 1000)	N/A	σ_k_ ~ U(0,10)
Arm-synthesis model 2^e^ (ASM2)	Arm-based model and likelihood	pcnetmeta (version 2.4)	Arm-level	Bayesian	Binomial likelihood and probit link	Heterogeneous	μ_k_~ N(0, 1000)	N/A	σ_bk_ ~ U(0,10)

Abbreviation: N/A, not applicable.

a Source: documentation for **gemtc** package https://cran.r-project.org/package=gemtc.

b τ_bk_ represents the between-trial heterogeneity standard deviation in treatment effects of treatment k relative to the baseline b.

c Each network was categorized according to the type of its included treatment comparisons and outcomes.^15^ Specifically, in the presence of placebo in the network, the network was categorized as pharmacological vs. placebo. If only pharmacological treatments were available then the network was categorized as pharmacological vs. pharmacological, whereas if a non-pharmacological treatment was included in the network, then the network was categorized as non-pharmacological vs. any category. Outcomes were categorized as all-cause mortality, subjective or semi-objective. The predictive distributions for between-trial heterogeneity variance for each of the treatment comparison by outcome type categories, estimated in Turner et al.,^15^ were used as informative priors.

d Model assumes homogeneity of the variances (i.e., common variance) of the random effects and assumes that the off-diagonal elements of the correlation matrix are equal (specified by the **hom_eqcor** option in **pcnetmeta** package).

e Model assumes an unstructured covariance matrix of the random effects and assumes that the off-diagonal elements of the correlation matrix are equal (specified by the **het_eqcor** option in **pcnetmeta** package).

The CSM1 and CSM2 models were fitted using R statistical software,^25^ specifically the R package **gemtc**, CSM3 was fitted using the R package **netmeta** ^26^ and ASM1 and ASM2 were fitted using the R package **pcnetmeta** ^27^ with the hom_eqcor and het_eqcor options, respectively. One of the datasets that met our eligibility criteria is provided in a supplementary file (note that the file is a Stata version 18 datafile), and the R code to fit the models is also provided in the Supplementary Material (pp. 43–47).

### 2.4 Fitting of Bayesian models and assessment of convergence

We used three chains with a burn-in of 300,000 (for CSM1 and CSM2) and 150,000 (for ASM1 and ASM2) followed by 300,000 samples saved at an interval of 10 from each of the three chains.^28^ We assessed the convergence using the Brooks–Gelman–Rubin method^29^ ^,^ ^30^ and by visual inspection of the history plots. For CSM1 and CSM2, convergence was assessed for each direct comparison and for 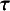 by visually inspecting the trace plot, posterior kernel density plot, shrink plot, and shrink factor. For ASM1 and ASM2, we assessed convergence of direct and indirect comparisons by visually inspecting the trace plot and shrinkage factors for the treatment comparisons. When the contrast between a pair of treatments within a network failed to converge, we first re-ran the model and saved samples at an interval of 500 from each of the three chains. If we were still unable to achieve convergence, the network was removed from subsequent analyses.

### 2.5 Network estimates

Prior to analyzing the networks, we standardized the direction of the outcomes so that an event indicated benefit. For harm outcomes, this meant we switched the events and non-events.

After analyzing each network using the five synthesis models described above, we estimated 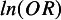 and their corresponding standard errors 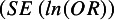 for each treatment compared with control/placebo (i.e., for a network with three treatments (1, 2, 3), where 3 was control/placebo, the included network estimates were 1 vs. 3 and 2 vs. 3); the rank of each treatment based on the SUCRA^31^; the corresponding SUCRA value (or equivalently *P*-score^32^ for the CSM3), expressed as %; and 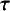 for the CSMs.

### 2.6 Differences in the network estimates between the methods

We used the following metrics to compare the network estimates (described in the ‘Network estimates’ section above) between the five synthesis models:

1. Difference in 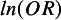

2. Ratio of 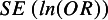

3. Difference in rank based on the SUCRA value
4. Difference in SUCRA value
5. Ratio of the estimates of the between-trial heterogeneity standard-deviation ( 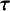 ) for the CSMs.

### 2.7 Exploring factors that might modify the agreements in network estimates between the synthesis models

The differences in the estimates between the synthesis models might be modified by several factors. We investigated the following metrics, the first three of which provide different ways of measuring the connectedness of the network:

1. The ratio of the number of treatments to the number of studies. Larger values indicate that there are many treatments for each study in the network and typically indicate well- connected networks. For example, network 481766 has a ratio of 0.88 whereas network 501348 has a ratio of 0.04 (Supplementary Table S2 and http://nma.emilykarahalios.com/?network=481766 and http://nma.emilykarahalios.com/?network=501348).
2. The ratio of the number of treatments to the number of *unique* direct comparisons. For example, suppose a network includes 3 studies where studies A and B compare treatments 1, 2, 3, and study C compares treatments 1, 2; this yields a total of 7 direct comparisons, but only 3 *unique* direct comparisons (i.e., 1 vs. 2, 1 vs. 3, and 2 vs. 3). Larger values of this ratio indicate networks with few treatments and poor connectedness (e.g., network 501371 has a value of 1.33), whereas small values indicate many treatments and a well-connected network (e.g., network 481378 has a value of 0.38) (Supplementary Table S2 and http://nma.emilykarahalios.com/?network=501371 and http://nma.emilykarahalios.com/?network=481378).
3. The ratio of the number of studies to the number of unique direct comparisons. Larger values indicate a larger number of studies per direct comparison and correspond to well-connected networks. For example, network 501348 has a value of 17.8, whereas network 501253 has a value of 0.68) (Supplementary Table S2 and http://nma.emilykarahalios.com/?network=501348 and http://nma.emilykarahalios.com/?network=501253).
4. The proportion of treatment arms in a network with fewer than 10 events. Larger values indicate a network with more sparse data. For example, network 480804 has a value of 0.66 whereas 476033 has a value of 0 (Supplementary Table S2 and (http://nma.emilykarahalios.com/?network=480804 and (http://nma.emilykarahalios.com/?network=476033). Note that this is not an indicator of the network being sparse (i.e., poorly connected).

### 2.8 Graphical methods

We generated graphical displays which we described previously.^20^ In brief, we graphed the estimates of the 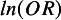 and 95% confidence/credible interval for each comparison from each of the networks. We used Bland–Altman plots to assess the pairwise agreement between the estimates ( 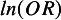 , 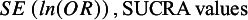 ) using the five synthesis models outlined above.^33^ For each pairwise comparison, the difference in the two estimates was plotted against the average of the two estimates. Regression-based limits of agreement (95%) were calculated and overlaid on the plots. To compare ranks, we graphically displayed the agreement between the ranks obtained from each method as a proportion of the total number of treatments for each rank.

### 2.9 Statistical analysis

To estimate differences in 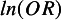 and 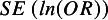 between the synthesis models, we fitted multilevel models with fixed effects for the synthesis model, random intercepts for network and treatment comparison within each network, and allowed the residual variance to vary across the models. These multilevel models were fitted to the treatment estimates, comparing each treatment to the reference treatment (i.e., control/placebo treatment). For example, for a network with three treatments, 1, 2, 3, where 1 was the control/placebo arm, we included estimates comparing treatments 2 vs. 1, and 3 vs. 1. These models did not account for uncertainty in estimating 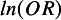 and 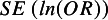 . To estimate the differences in the rank and SUCRA (%) values between the synthesis models, we again fitted multilevel models with random intercepts for the network. For these multilevel models, we selected the treatment ranked highest (i.e., rank = 1) for each synthesis model and kept the corresponding ranking and SUCRA value from the other synthesis models. For the multilevel model for SURCRA values, we allowed the residual variance to vary across the models. Finally, for the CSMs, we fitted a linear regression model to estimate differences in 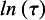 between the synthesis models. This regression did not account for uncertainty in estimating 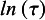 .

The initial regression models included only terms for the synthesis models used to synthesize the data. CSM1 (i.e., CSM in a Bayesian framework with a non-informative prior) was chosen as the reference synthesis model. Additional models included interaction terms between the synthesis model and the factors that we hypothesized to modify the differences in the network estimates between the synthesis models (note that a separate model was fitted for each factor). Including the interaction terms allowed the linear relationship between the continuous factor and the network estimates to vary by synthesis model. From these additional multilevel models, and for each synthesis model, we calculated the *difference* between the predicted network estimates calculated at the 75th and 25th percentiles of the hypothesized factor of interest. We then compared the *difference* for each synthesis model against the *difference* for the reference model (i.e., CSM2 vs. CSM1, etc.). For outcomes on the log scale, we exponentiated the differences, which yielded ratios. See Supplementary Material (pp. 5–6) for the model and formulae.

Stata version 18^34^ was used to fit the multilevel models and to create the graphical displays.

## 3 Results

**Figure 1:**
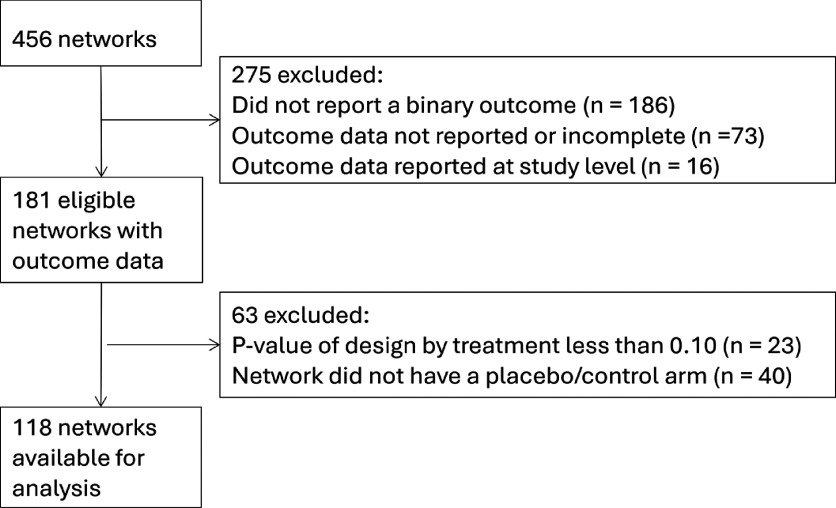
Flow diagram of networks included in the empirical analysis comparing contrast-synthesis and arm-synthesis models to synthesize evidence in network meta-analysis.

Of the 456 available networks, 118 were available for our analyses (see Figure 1 for reasons for exclusion).

### 3.1 Characteristics of included networks

The list of networks and their characteristics are provided in Supplementary Table S2, the network plots are available as Online material http://nma.emilykarahalios.com/ and summary characteristics are provided in Supplementary Table S3. The median number of included treatments was 7 (inter-quartile range (IQR): 5, 9). The median number of studies included in each network was 20 (IQR: 13, 40). Of the 118 networks, 31 (26%) were star-shaped. Most networks compared a pharmacological treatment to placebo (n = 101; 86%), 4 (3%) compared pharmacological vs. pharmacological treatments, and 13 (11%) included at least one non-pharmacological treatment. Most of the networks had an objective (*n* = 43; 36%) or semi-objective (*n* = 52; 44%) outcome; only 23 (20%) had a subjective outcome.

### 3.2 Results of fitting Bayesian models and assessment of convergence

The convergence and estimation results from fitting the models to the networks are provided in the Supplementary Material (pp. 18–26), characteristics of the 25 networks that failed to converge after inspection of the convergence diagnostics when fitting ASM2 are provided in Supplementary Table S4, and the characteristics of the three networks that failed to yield one or more estimates (i.e., 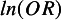 , or 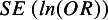 ) using one of the ASMs are provided in Supplementary Table S5.

### 3.3 Comparison of the contrast-synthesis and arm-synthesis models

#### 3.3.1 Odds ratios

**Figure 2:**
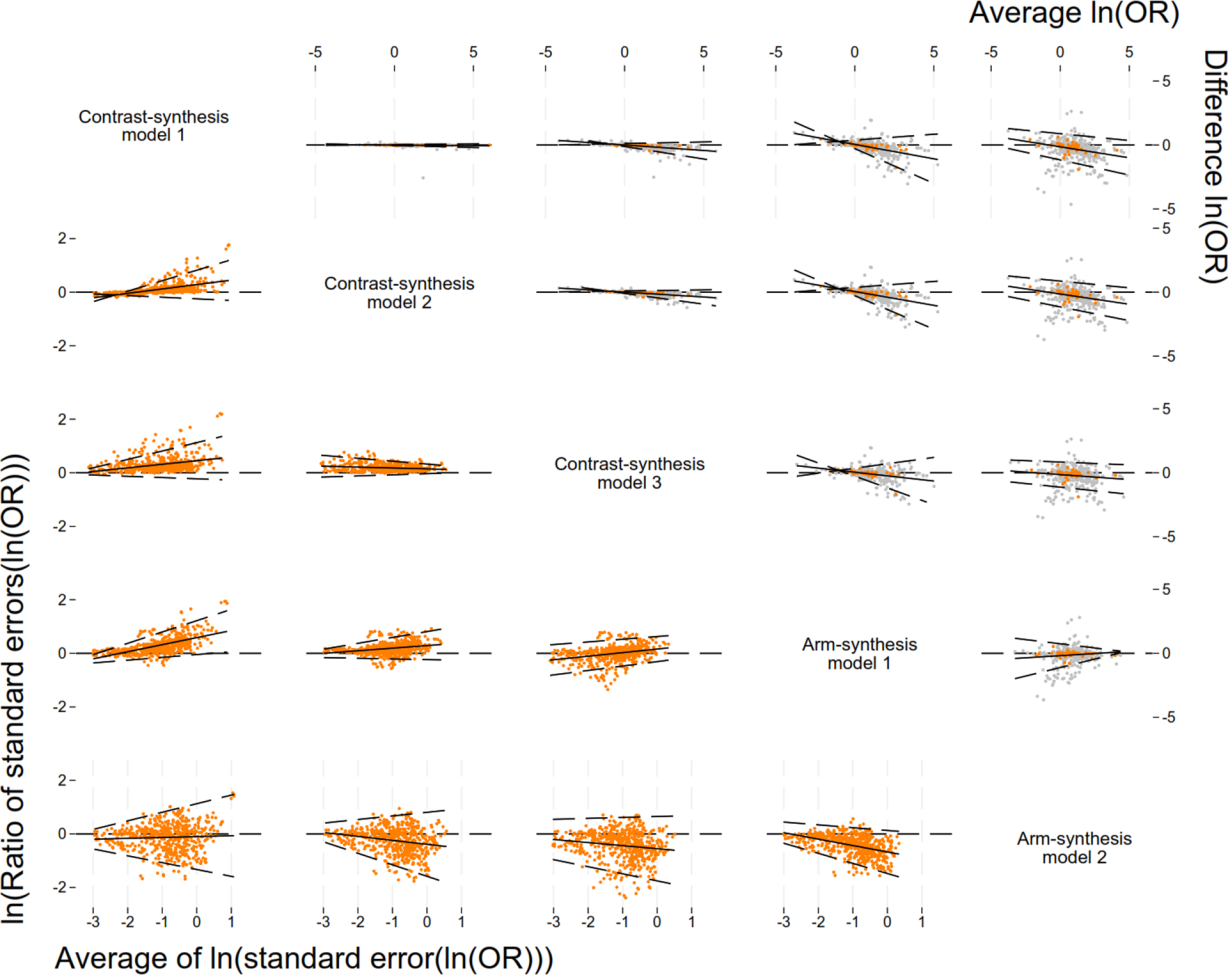
Bland-Altman plots for the level of agreement between the log of the odds ratios (top right) and standard errors for the log of the odds ratios (bottom left) comparing the five models used to synthesise evidence from a network meta-analysis. Plots in the top triangle show the difference in the ln(odds ratio (OR)) (row method – column method) on the vertical axis and average of the ln(OR) on the horizontal axis. For each network, we present the ln(OR) for the treatment comparison compared with control/placebo in grey, and the median ln(OR) from each network in orange. Plots in the bottom triangle show the differences in ln(standard errors) on the vertical axis (ln(ratio of standard errors)) (column method – row method) and the average of the ln(standard errors) on the horizontal axis. For each network, we present the ln(standard errors) for each treatment comparison in orange. Black solid lines indicate the average, black dashed lines indicate the 95% regression based limits of agreement. Abbreviations: ln, natural logarithm; OR, odds ratio. Note that in the Bland-Altman plots, the negative slopes, as observed for the ln(OR), indicate that the row model is further from 0 than the column model when both are negative, and nearer to 0 when both are positive; i.e., that the row model is more conservative.

The estimates obtained from the two CSMs implemented in a Bayesian framework (i.e., CSM1 and CSM2) using data from 118 networks with 867 treatment comparisons were similar (ratio of 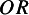 s comparing CSM2 vs. CSM1: 1.03 (95% Confidence Interval (CI): 0.72, 1.46)) (Table 2 – 3rd column; Dataset available in Excel file—Supplementary Table S12). The estimates obtained from the CSM implemented in a frequentist framework (i.e., CSM3) tended to show less benefit than those obtained from the Bayesian CSMs: CSM3 vs. CSM1: 0.57 (95% CI: 0.44, 0.73), and CSM3 vs. CSM2: 0.55 (95% CI: 0.43, 0.72) (Table 2 – 4th column). The Bland-Altman plots (Figure 2) reflected these results. Given that CSM1 and CSM2 were similar, we focus on the comparison of CSM3 vs. CSM1. The corresponding Bland-Altman plot (Figure 2) showed that the difference in the 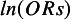 depended slightly on the size of the 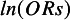 , but the regression-based limits of agreement were narrow (ranging from –0.2 to 0.2).

**Table 2:** Comparison of the ratio of the odds ratios across the five synthesis models

	Contrast-synthesis	Contrast-synthesis	Contrast-synthesis	Arm-synthesis	Arm-synthesis
	model 1	model 2	model 3	model 1	model 2
No. of networks (no. of					
treatment					
comparisons)	118 (867)	118 (867)	118 (867)	116 (853)	90 (557)
Contrast-synthesis model 1	Ref	1.027 (0.720, 1.463)	0.568 (0.444, 0.725)	0.694 (0.508, 0.949)	0.473 (0.370, 0.606)
		–	*p* = 0.885	*p* < 0.001	*p* = 0.022	*p* < 0.001
Contrast-synthesis model 2		Ref	0.553 (0.426, 0.717)	0.676 (0.489, 0.935)	0.461 (0.355, 0.599)
			–	*p* < 0.001	*p* = 0.018	*p* < 0.001
Contrast-synthesis model 3			Ref	1.223 (1.003, 1.493)	0.834 (0.791, 0.879)
				–	*p* = 0.047	*p* < 0.001
Arm-synthesis model 1				Ref	0.682 (0.557, 0.833)
					–	*p* < 0.001

The table shows the ratio of odds ratios (95% confidence interval) and corresponding *p*-value (column model compared to row model). For example, the ratio of odds ratios comparing arm-synthesis model 2 to arm-synthesis model 1 (includes 557 treatment comparisons from 90 networks) is 0.68 (95% CI: 0.56, 0.83), which indicates that the odds ratios from arm-synthesis model 2 are, on average, 32% smaller than the odds ratios from arm-synthesis model 1.

For ASM1, the estimated 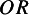 s showed less benefit than those estimated from the Bayesian CSMs (using data from 116 networks with 853 treatment comparisons) (ratio of 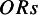 , ASM1 vs. CSM1: 0.69 (95% CI: 0.51, 0.95), ASM1 vs. CSM2: 0.68 (95% CI: 0.49, 0.94), whereas they showed more benefit than those estimated from the frequentist CSM (ASM1 vs. CSM3: 1.22 (95% CI: 1.00, 1.49)) (Table 2 – 5th column). The Bland-Altman plots (Figure 2) showed that the difference in 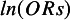 between the methods depended on the size of the 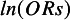 and the 95% regression-based limits of agreement were wide. For example, for the comparison between ASM1 and CSM1, at the midpoint of the 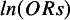 , the limits of agreement ranged from –0.7 to 0.4 meaning that one odds ratio in 20 was estimated to be more than 1.5 times larger for CSM1 compared to ASM1.

For ASM2, the estimated 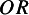 s showed less benefit than those estimated from the CSMs and ASM1 (using data from 90 networks with 557 treatment comparisons) (Table 2 – 6th column). The Bland-Altman plots (Figure 2) showed large variability in the differences in 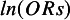 , which varied according to the size of the 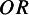 and resulted in wide 95% regression-based limits of agreement. For example, for the comparison between ASM2 and CSM1, at the midpoint of the 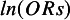 , the limits of agreement ranged from –1.3 to 0.8.

#### 3.3.2 Standard errors of the natural logarithm of the odds ratio

We compared the 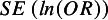 (Table 3 and Figure 2 – lower triangle) and found that the main differences between the CSM models arose in the comparison of CSM3 to CSM1 and CSM2. The standard errors for CSM3 were, on average, smaller than those obtained from CSM1 (ratio of 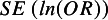 : 0.70; 95% CI: 0.67, 0.72) and CSM2 (ratio of 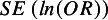 : 0.77; 95% CI: 0.75, 0.79) (Table 3). For the comparison of CSM3 to CSM1, the Bland-Altman plot (Figure 2 – row 3/column 1) showed that the ratio was dependent on the size of the 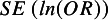 and the limits of agreement were wide for large standard errors. For the comparison of CSM3 to CSM2, the Bland-Altman plot (Figure 2 – row 3 / column 2) showed little variability across the standard errors.

**Table 3:** Comparison of the ratio of the standard error of the logarithm of the odds ratio across the five synthesis models

	Contrast-synthesis	Contrast-synthesis	Contrast-synthesis	Arm-synthesis	Arm-synthesis
	model 1	model 2	model 3	model 1	model 2
No. of networks (no. of treatment					
comparisons)	118 (867)	118 (867)	118 (867)	116 (853)	90 (557)
Contrast-synthesis model 1	Ref	0.903 (0.890, 0.917)	0.695 (0.674, 0.717)	0.694 (0.675, 0.713)	1.139 (1.094, 1.187)
		–	*p* < 0.001	*p* < 0.001	*p* < 0.001	*p* < 0.001
Contrast-synthesis model 2		Ref	0.769 (0.747, 0.792)	0.768 (0.748, 0.788)	1.261 (1.212, 1.313)
			–	*p* < 0.001	*p* < 0.001	*p* < 0.001
Contrast-synthesis model 3			Ref	0.998 (0.962, 1.036)	1.639 (1.562, 1.720)
				–	*p* = 0.930	*p* < 0.001
Arm-synthesis model 1				Ref	1.642 (1.568, 1.719)
					–	*p* < 0.001

The table shows values for the ratio of the standard error of the logarithm of the odds ratios (95% confidence interval) and corresponding *p*-value (column model compared to row model). For example, the ratio of the standard error of the logarithm of the odds ratios comparing arm-synthesis model 2 to arm-synthesis model 1 (includes 557 treatment comparisons from 90 networks) is 1.64 (95% CI: 1.57, 1.72), which indicates that the standard errors of the logarithm of the odds ratio for arm-synthesis model 2 are, on average, 64% larger than the standard errors from arm-synthesis model 1.

Comparing the 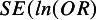 between the ASM models, we found that ASM2 yielded larger 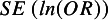 (ratio of 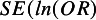 ): 1.64; 95% CI: 1.57, 1.72), which were more different than the differences observed between the CSM models. Comparing ASM1 to CSM1, smaller standard errors were observed (ratio of 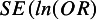 ): 0.69; 95% CI: 0.68, 0.71). The Bland-Altman plot (Figure 2 – lower triangle) showed that the ratios were dependent on the size of the 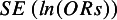 and the limits of agreement were wide.

#### 3.3.3 Treatment ranks and Surface Under the Cumulative RAnking curve (SUCRA) values

**Figure 3:**
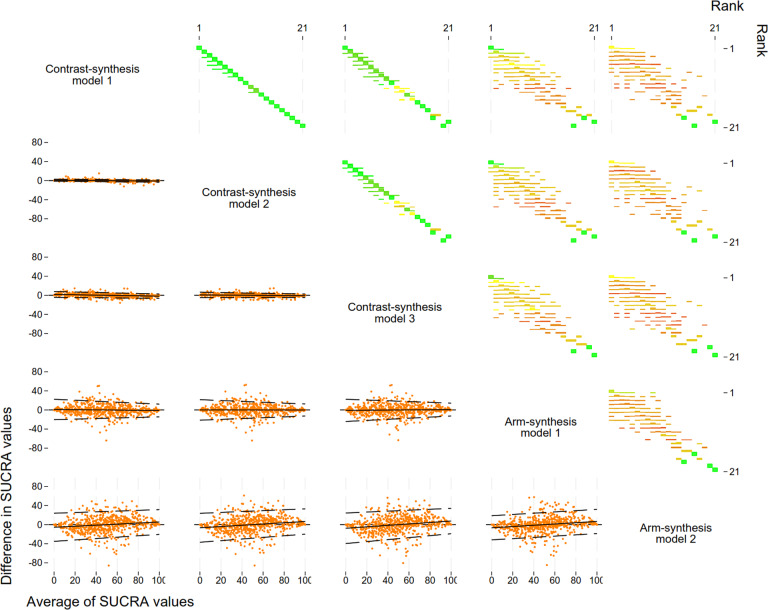
Comparison of ranks (top right) and Bland-Altman plots for the level of agreement between the SUCRA values (bottom left) obtained from the five models used to synthesise evidence from a network meta-analysis. Plots in the top triangle show the agreement between the ranks obtained from each method as a proportion of the total number of treatments for each rank. Plots in the bottom triangle show the differences in SUCRA values on the vertical axis (column method – row method) and the average of the SUCRA values on the horizontal axis. Black solid lines indicate the average, black dashed lines indicate the 95% regression-based limits of agreement. Abbreviation: SUCRA, surface under the cumulative ranking.

The results of our investigation of agreement between the models were similar when examining treatment ranks derived using the SUCRA values / P-score and the SUCRA values themselves; here we report the results from the treatment ranks (Table 4 and Figure 3; see Supplementary Table S6 for results of the SUCRA values). We observed excellent agreement between the CSMs in terms of treatment ranks. For example, when selecting the treatment ranked as 1 using CSM1 and comparing the resulting rank from CSM2 and CSM3, on average, we observed an average change in rank of less than 0.06 units (Table 4 – row 2/columns 2–4).

**Table 4:** Comparison of the difference in the treatment ranks based on the Surface Under the Cumulative RAnking curve (SUCRA) or P-score between the synthesis models (column model compared to row model) after selecting the treatment ranked as 1 (i.e., the treatment with the highest SUCRA / P-score value) for the model in the row and retaining the corresponding SUCRA/P-score from the other synthesis models (columns)^a^

Rank 1 obtained from					
model below no. of networks	Contrast-synthesis	Contrast-synthesis	Contrast-synthesis	Arm-synthesis	Arm-synthesis
(no. of observations)	model 1	model 2	model 3^b^	model 1	model 2
Contrast-synthesis model 1	Ref	0.000 (–0.000, 0.000)	0.051 (0.011, 0.091)	0.207 (0.104, 0.310)	0.559 (0.354, 0.765)
118 (560)	–	*p* = 1.000	*p* = 0.012	*p* < 0.001	*p* < 0.001
Contrast-synthesis model 2	–0.000 (–0.000, 0.000)	Ref	0.051 (0.011, 0.091)	0.207 (0.104, 0.310)	0.559 (0.354, 0.765)
118 (560)	*p* = 1.000	–	*p* = 0.012	*p* < 0.001	*p* < 0.001
Contrast-synthesis model 3	0.051 (0.011, 0.091)	0.051 (0.011, 0.091)	Ref	0.215 (0.109, 0.322)	0.596 (0.389, 0.803)
118 (560)	*p* = 0.012	*p* = 0.012	–	*p* < 0.001	*p* < 0.001
Arm-synthesis model 1	0.414 (0.029, 0.799)	0.405 (0.022, 0.788)	0.362 (0.071, 0.653)	Ref	0.785 (0.436, 1.134)
116 (554)	*p* = 0.035	*p* = 0.038	*p* = 0.015	–	*p* < 0.001
Arm-synthesis model 2	0.767 (0.458, 1.075)	0.767 (0.458, 1.075)	0.789 (0.486, 1.092)	0.744 (0.417, 1.072)	Ref
90 (450)	*p* < 0.001	*p* < 0.001	*p* < 0.001	*p* < 0.001	–

The table shows value for the difference in the ranks (95% confidence interval) and corresponding *p*-value.

a For example, if we keep the treatments ranked as 1 using arm-synthesis model 2 (last row) and compare the ranks to contrast-synthesis model 1 (2nd column), we see that, on average, the ranks obtained from contrast-synthesis model 1 are 0.8 units higher (95% CI: 0.5, 1.1) than those obtained from arm-synthesis model 2.

b Note that this corresponds to the p-score for contrast-synthesis model 3.

We observed small variability in the treatment ranks between ASM1 and the CSMs. This was consistent when we selected the treatment ranked as 1 from each of the CSMs (Table 4 – rows 2–4/column 5) and when we selected the treatment ranked as 1 from the ASM1 (Table 4 – row 5/columns 2–4). There were much larger differences, on average, in the treatment ranks when comparing ASM2 to the other synthesis models. This was consistent when we selected the treatment ranked as 1 from ASM2 (Table 4 – row 6/columns 2–5). We also observed the largest variabilities in the differences in treatment ranks when comparing the ranks obtained from ASM2 to those obtained from the other synthesis models (Figure 3 – column 5 top triangle and row 5 bottom triangle). However, the difference in treatment ranks was less than 1 unit when comparing all models to each other.

#### 3.3.4 Between-study heterogeneity

The between-study heterogeneity standard deviation of the relative treatment effects ( 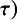 (estimated from 118 networks for CSM1, CSM2 and CSM3) was, on average, similar for the CSM2 compared to CSM1 (ratio of 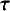 : 0.88; 95% CI: 0.63, 1.23), but much smaller for CSM3 compared to both CSM1 (ratio of 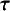 : 0.22; 95% CI: 0.16, 0.31) and CSM2 (ratio of 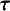 : 0.25; 95% CI: 0.18, 0.35) (Table 5).

**Table 5:** Comparison of the ratio of the square root of the between-study heterogeneity variance (τ)

	Contrast-synthesis	Contrast-synthesis	Contrast-synthesis
	model 1	model 2	model 3
No. of networks	118	118	118
Contrast-synthesis model 1	Ref	0.879 (0.629, 1.227)	0.222 (0.159, 0.310)
		–	*p* = 0.447	*p* < 0.001
Contrast-synthesis model 2		Ref	0.253 (0.181, 0.353)
			–	*p* < 0.001

The table shows the value for the ratio of the square root of the between-study heterogeneity variance.

(95% confidence interval) and corresponding *p*-value (column model compared to row model).

### 3.4 Factors that modify the ratio of the metrics between the models

Of the four factors that we hypothesized might modify differences in the network estimates between the synthesis models, three factors were found to do so (the ratio of the number of treatments to the number of studies, ratio of the number of studies to the number of unique direct comparisons, and the proportion of arms in the network with fewer than 10 events) for the 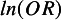 (Supplementary Material [p. 28] and Supplementary Tables S7). For these factors, the estimates obtained from CSM2 were similar to those obtained from CSM1. For the ratio of the number of treatments to the number of studies and the proportion of arms in the network with fewer than 10 events, the estimates obtained from CSM3, ASM1 and ASM2 showed less benefit than for CSM1 and CSM2 (Supplementary Tables S7a and S7d). On the other hand, for the ratio of the number of studies to the number of unique direct comparisons, the estimates obtained from CSM3, ASM1 and ASM2 showed more benefit than for CSM1 and CSM2 (Supplementary Tables S7c). For the standard errors and SUCRA values, all the factors were found to modify the estimates (Supplementary Material pages 32, 35 and Supplementary Tables S8 and S9), whereas only two of the factors (ratio of the number of treatments to the number of studies, and ratio of the number of studies to the number of direct comparisons) were found to modify the difference in the treatment ranks (Supplementary Table S10). None of the factors were found to modify the differences in estimates of between-study standard deviation between the synthesis models (Supplementary Material [p. 40] and Supplementary Table S11).

## 4 Discussion

To the best of our knowledge, this is the first empirical study to compare contrast- and arm-synthesis NMA models applied to a large number of networks with binary outcomes. In general, we found good agreement between the CSMs. CSM3 yielded odds ratios that showed less benefit, and standard errors and values of 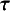 that were smaller than those obtained from CSM1 and CSM2. The smaller values of 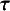 are likely contributing to the smaller values of the standard errors. Further, the SUCRA values and treatment ranks from the CSMs were in close agreement. ASM1 had poor average agreement with all CSMs (the odds ratios showed greater benefit; the standard errors were smaller; and the treatment ranks were slightly lower than the CSMs) and a large amount of variability was observed in the differences of all the aforementioned metrics. Notably, these differences were observed for the comparison between ASM1 and CSM1, which is considered the most direct comparison to synthesis models that are commonly used. Finally, ASM2 had poor average agreement with the CSMs and ASM1 (the ORs showed less benefit, the standard errors were larger, the SUCRA values were much lower, and the resulting treatment rank was larger, corresponding to a lower rank, compared with the three CSMs and ASM1) and, like ASM1, there was large variability in the differences of all metrics. Of note, a major limitation of fitting the ASMs particularly ASM2, is that they can fail to yield estimates or converge (in our study, this occurred for 28/118 networks).

Of the four factors that were hypothesized to predict the differences in network estimates between the models, a number of associations were observed. For example, the ‘ratio of the number of studies to the number of direct comparisons’, was associated with differences in the estimates of the standard errors, SUCRA and rank but not the odds ratios. Whereas the proportion of arms with fewer than 10 events was associated with differences in the odds ratios and the standard errors.

### 4.1 Comparison with previous research and explanation of the study findings

As expected, and as found by Seide et al.,^35^ the estimates of the 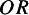 s obtained from the CSMs in a Bayesian framework with non-informative (CSM1) and informative (CSM2) priors were similar, and the estimates for standard errors from CSM2 were, on average, smaller. The use of an informative prior will increase the standard error when there is minimal heterogeneity present but reduce the standard error in the presence of a large amount of heterogeneity. Similar to the findings of Seide et al.,^35^ the differences in the standard errors became smaller as the networks became less sparse.

Previous case studies have found that fitting a CSM in a frequentist framework (i.e., CSM3) will yield similar odds ratios compared to fitting a CSM in a Bayesian framework (i.e., CSM1 and CSM2).^4^ However, similar to the findings of Seide et al.,^35^ we found that the odds ratios obtained from CSM3 showed less benefit (i.e., were nearer to 1) than those obtained from CSM1 and CSM2. Standard errors obtained from Bayesian models are generally larger than those obtained from frequentist models,^35^ and this held true when comparing the standard errors from CSM3 to those obtained from CSM1 and CSM2.

We observed differences between the CSMs and ASMs. Of note, the differences in the magnitude of the 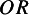 s and the standard errors depended on which ASM was fitted to the network. In CSMs, the study intercepts are fixed effects (see White et al.^13^ for further explanation), which means that the 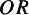 s are estimated using only the differences within studies. Conversely, in the ASMs, the treatment effects are estimated using both within-study information and the differences between studies; this extra between-study information could be causing the differences in the estimates of the 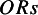 between the models. Further, although we limited our study by removing networks that showed evidence of inconsistency, the failure to detect inconsistency using a statistical test does not mean that there is consistency in the network and that the transitivity assumption has not been violated.^36^ When consistency and the transitivity assumption are violated, differences between the estimates obtained from the CSMs and ASMs can arise. Theoretically, the ASMs should reduce the extent of the bias in this circumstance.^13^ Finally, the CSMs yield conditional estimates of the treatment effect, whereas the ASMs yield marginal estimates, which may also explain the differences.

Contrary to what we expected, the two ASMs that were fitted did not yield similar estimates. The main difference between these two models was in the assumption of the variance of the 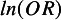 , where ASM1 assumes the variance is the same across different treatments (i.e., homogeneity).

### 4.2 Strengths and limitations

There are several strengths of our study. We defined our methods in a published protocol.^20^ We used a large number of networks to compare the methods, which allowed us to conclude with reasonable confidence that, for those methods evaluated, the choice of NMA method can yield large differences in the 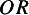 , standard error, SUCRA value, treatment rank and between study heterogeneity. Further, we evaluated more than one model within each approach and limited the dataset to networks where there was no evidence of statistical inconsistency (using the design by treatment interaction test), which ensured a fairer comparison across the models.

However, we focused on NMAs with binary primary outcomes, so our findings might not be generalizable to other outcome types (e.g., continuous outcomes). We excluded 28 networks from the evaluation of the ASMs. Reasons for this include: three networks failed to produce output, which might have arisen because of the packages that we used to fit the models, rather than a problem with the arm-synthesis approach. However, we chose to use available packages to fit the models and recognize this limitation of our analysis. For an additional 25 networks, we observed issues with the convergence diagnostics. Due to the nature of the ASMs (i.e., that the absolute estimates of each arm are pooled and population-averaged treatment effects are constructed from the arm estimates), convergence of the Markov Chain Monte Carlo algorithms is slower than the CSM, and the estimates may not converge if some treatments only included in a few studies.^27^

While we examined five models (three CSMs and two ASMs), other models have been proposed that we have not evaluated or discussed (e.g., Thorlund et al.^4^ and Sauter et al.^37^). Further, in our evaluation of CSM2, we used the between-trial heterogeneity variance priors from Turner et al.^15^ and assumed equal heterogeneity across the network. For a given network, there may be a set of available priors (e.g., networks including pharmacological and non-pharmacological interventions) and we selected the prior that had the largest between-trial heterogeneity variance. Because we limited our networks to those that had a control/placebo arm, the majority (86%) of our networks were classified as ‘pharmacological vs. placebo’. We chose this approach because it is conservative but assigning between-trial heterogeneity variance with the largest magnitude and assuming equal heterogeneity across the network is a strong assumption, which might not be plausible, and other choices might impact the findings.^38^

There are a range of metrics that could be compared across the models. For example, we examined the 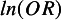 and its corresponding standard error, treatment rank based on the SUCRA value, the SUCRA value and the between-trial heterogeneity variance, but other metrics (e.g., risk ratio) could be examined.^39^ Further, to assess agreement between the treatment hierarchies across the synthesis models, we compared the difference in the ranks and SUCRA values. However, these metrics have limitations; particularly for networks with many treatments, small differences in the SUCRA values could be important and lead to differences in treatment ranks, whereas in networks with few treatments, large differences in SUCRA values are required to change the treatment ranks.^40^ As well, ranking SUCRAs is not a standard way to rank treatments.^41^ Finally, we had originally planned to compare the estimates for 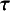 for the CSMs, but we did not plan to extract or calculate the estimates from the ASMs.

We fitted the CSMs using the **gemtc** (Bayesian), and **netmeta** (frequentist) packages in R. However, the CSMs could also be fitted using, for example, the **multinma** package^42^ (Bayesian) in R or the **network** package^23^ in Stata. To our knowledge, there are no other packages available in R to fit the ASMs. We have tried to be explicit in the model specifications to ensure that a reader choosing to implement these models using a different package could fit the same model, and, in theory, produce the same results. However, we did not set out to investigate whether the same models fitted using different packages would yield similar results.

### 4.3 Implications for analysts

CSM1 and CSM2, on average, yielded similar estimates of treatment effects, standard errors, and treatment ranks. Given this, the choice between these models in practice may often be of limited consequence. However, this is not true for choosing between CSM and ASM, where the estimates of treatment effects and standard errors can be very different. In particular, the ASMs can yield standard errors that are larger or smaller than those obtained from the CSMs depending on the chosen model. Further, the SUCRA values and corresponding treatment ranks obtained from ASM2 were different from those obtained from the other models, which can have an impact on the final ranking of the treatment options compared. Given these differences, analysts need to ensure they pre-specify their model and provide a justification for their choice. Use of random study intercepts in an ASM should be avoided unless it can be justified explicitly, because this model compromises randomization and may introduce bias.^13^

### 4.4 Future research

In our study, we have examined empirically the impact of applying the methods in practice. Future research that examines the performance of the models using statistical simulation would be helpful. Empirical evaluations and simulation studies could examine other summary estimates that can be obtained from trials with binary outcomes (e.g., risk ratio); focus on networks with continuous or time-to-event outcomes; allow the prior distributions to vary across the networks; allow the between-study heterogeneity to vary across the treatment comparisons (e.g., as proposed by Thorlund et al.^4^); use fixed instead of random study effects when fitting ASMs^9^; investigate a newer method proposed to improve the estimates obtained from ASMs^43^; assess the impact of using other models that have been proposed (e.g., Sauter et al.^37^); and assess the impact of the different models to assess inconsistency.^44^ ^–^ ^46^ Furthermore, recent discussion suggests that ASM and CSM are likely to be equivalent^6^ ^–^ ^9^ when the study effects in the ASM are assumed to be fixed rather than random (note that the CSM model can be extended to allow for the variance to depend on the treatment, but this is more conveniently modelled by the ASMs); comparison of these models would be useful, in particular focusing on variances of the relative treatment effects.^6^ Finally, variance heterogeneity for sparse comparisons is usually more conveniently handled in a Bayesian approach which shrinks the sparse evidence using a mildly informative prior – further investigation of these models is warranted.

## 5 Conclusion

We found that different models used to synthesize evidence in an NMA can yield different estimates of odds ratios and standard errors, leading to differing SUCRA values that can have an impact on the final ranking of the treatment options compared. Further work is needed to provide a more comprehensive understanding of when these models differ and if recent advances in the implementation of the ASMs will mitigate these differences. Analysts undertaking NMA should ensure that the model is specified *a priori.*

## Supporting information

Karahalios et al. supplementary material Karahalios et al. supplementary material

## Data Availability

The authors confirm that the data supporting the findings of this study are available within the article’s Supplementary Material.

## Supplementary material

To view supplementary material for this article, please visit http://doi.org/10.1017/rsm.2025.18.
