# Supplementary material for: An investigation of the impact of using contrast- and arm-synthesis models for network meta-analysis
Source: Res Synth Methods. 2025 Apr 25;16(4):631–49. doi: 10.1017/rsm.2025.18 (PMC12527487; doi:10.1017/rsm.2025.18)
Supplement: Karahalios et al. supplementary material [file S1759287925000183sup001.zip › SuppMaterial_NMA empirical paper.pdf]

Supplementary Material

Contents

Supplementary Methods 3

Description of the mathematical models fitted to explore factors that might modify the differences in network estimates between the synthesis models. 5

Supplementary Results 7

Convergence and estimation 19

Factors that modify the ratio of the odds ratio between the models 29

Factors that modify the ratio of the standard errors between the models 32

Factors that modify the ratio of the SUCRA values and treatment ranks between the models 35

Factors that modify the ratio of the between-study heterogeneity between the models 40

R code to fit the models to one of the eligible datasets. 43

List of Tables

Supplementary Table S1. Deviations from the study protocol 3

Supplementary Table S2. Characteristics of the included networks 7

Supplementary Table S3. Summary characteristics of networks 18

Supplementary Table S4. Characteristics of networks and treatments that did not appear to converge for arm-synthesis model 2 after inspection of the convergence diagnostics. 19

Supplementary Table S5. Characteristics of networks that failed to run using one of the models...... 27

Supplementary Table S6. Comparison of the difference between the Surface Under the Cumulative RAnking curve values (SUCRA) (%) or P-score (%) between the synthesis models (comparing column model to row model) after selecting the treatment ranked as 1 (i.e. the treatment with the highest SUCRA / p-score value) for the model in the row and retaining the corresponding SUCRA/P-score from the other synthesis models [a]. 28

Supplementary Table S7. Results to assess whether the following factors modified the ratio of the odds ratio between the models. 30

Supplementary Table S8. Results to assess whether the factors modified the ratios of the SE(log(OR)) between the models.. 33

Supplementary Table S9. Results to assess whether the following factors modified the differences in the SUCRA values between the models. 36

Supplementary Table S10. Results to assess whether the following factors modified the differences in the ranks between the models... 38

Supplementary Table S11. Results to assess whether the following factors modified the differences in the square-root of the between study heterogeneity (⍰) between the models................................. 41

Supplementary Table S12. Data for the NMA empirical paper. 42

Supplementary Methods

Supplementary Table S1. Deviations from the study protocol

Protocol method	Deviation from protocol method, with justification
We planned the following four graphical displays of the data to compare the network estimates between the methods:<br>1. Graph the log of the odds ratios and 95% confidence/credible interval estimated from each method for each comparison within each of the networks,<br>2. Bland-Altman plots will be presented to assess the agreement between the estimates of the log of the odds ratios and standard errors of the log of the odds ratios using the five methods,<br>3. Bland-Altman plots to assess the agreement between SUCRA estimates,<br>4. To compare ranks, we will graphically display the agreement between the ranks obtained from each method as a proportion of the total number of treatments for each rank	We added an additional graphical display to compare the estimates of the odds ratios (and 95% confidence/credible intervals) between each method. This additional plot includes the median estimate from each network.<br><br>*Type of deviation: addition*
We planned to include all treatment arms within each network.	We excluded treatments within networks that were disconnected.<br><br>*Type of deviation: modification*
To implement the Bayesian analyses we planned to use a burn-in of 300,000 for contrast-synthesis models 1, 2 and arm-synthesis models 1, 2.	We used a burn-in of 150,000 for arm-synthesis models 1 and 2.<br><br>*Type of deviation: modification*
We did not have a plan for how to deal with networks that did not yield estimates for all pairwise comparisons for a synthesis model.	We excluded the networks when comparing the synthesis model for which estimates were not obtained to the other methods.<br><br>*Type of deviation: modification*
We did not have a plan for dealing with treatment comparisons which did not converge (as assessed from inspection of the convergence diagnostics using contrast-synthesis models 1, 2 and arm-synthesis models 1, 2).	When convergence diagnostics indicated non-convergence, we re-ran the model and thinned every 500th sample (instead of every 10th sample). If convergence diagnostics still indicated problems, we excluded the network from comparisons with that synthesis model.

Protocol method	Deviation from protocol method, with justification
	Type of deviation: modification
To estimate the differences between the methods for the ranks and SUCRA values, we planned to fit multilevel models that included random effects for network and treatment comparison within the network.	To estimate the differences between the methods for the rank and SUCRA values, we selected the treatment ranked as 1 (i.e. the treatment with the largest SUCRA value) for each method and kept its corresponding ranking from the other methods. We then fitted multilevel models for rank and SUCRA values (%) for each method and included random effects for network.<br><br>*Type of deviation: modification*
To estimate the differences in $\boldsymbol{ln}(\boldsymbol{OR})$ and $\boldsymbol{SE}(\boldsymbol{ln}(\boldsymbol{OR}))$ between the synthesis models, we planned to fit multilevel models to all treatment comparisons from each network (e.g. for a network with three treatments A, B, C, we planned to include the $\boldsymbol{ln}(\boldsymbol{ORs})$ comparing treatments B vs A, B vs C, and C vs A).	To estimate the differences in $ln(OR)$ and $SE(ln(OR))$ between the synthesis models, we restricted our analyses to treatment effect estimates compared to the reference treatment (e.g., for a network with three treatments A, B, C, we included the ln(ORs) comparing treatments B vs A, and C vs A), thus ensuring that the comparisons are mathematically independent.<br><br>*Type of deviation: modification*
We planned to include all treatment comparisons for all networks that met the eligibility criteria. That is, i) the primary outcome of the network was binary, ii) the number of events and number of participants were available for each study-specific direct comparison within the network, and iii) for networks including at least one loop, there was no evidence of inconsistency in the network as detected by a p-value >0.10 via the design-by-treatment interaction test using the **mvmeta** command in Stata version 18.	We added a fourth inclusion criteria; to include only networks that had a control arm and ensured that the reference treatment in each network was a control treatment. This meant that the results from our models were more interpretable since they yielded an average difference between models of comparisons against control/placebo.<br><br>*Type of deviation: addition*

Description of the mathematical models fitted to explore factors that might modify the differences in network estimates between the synthesis models.

Below, we provide the formulae for the factor 'ratio of the number of treatments to the number of studies' (hence forth referred to as ratio). The factor can be substituted for the other factors described in the main and supplementary below manuscript.

Model fitted with the interaction term for the ratio and synthesis model:

$$\begin{aligned}\ln(OR)_{ij} = \beta_0 &+ \beta_1 \times CSM2_{ij} + \beta_2 \times CSM3_{ij} + \beta_3 \times ASM1_{ij} + \beta_4 \times ASM2_{ij} + \beta_5 \times ratio_{ij} \\ &+ \beta_6 \times CSM2 \times ratio + \beta_7 \times CSM3 \times ratio + \beta_8 \times ASM1 \times ratio \\ &+ \beta_9 \times ASM2 \times ratio \\ &+ u_i + v_{j(i)} + \epsilon_{ij}\end{aligned}$$

Where:

i = 1, …, 118 networks

j = 1, …, 45 treatments

CSM1 = contrast-synthesis model 1

CSM2 = contrast-synthesis model 2

CSM3 = contrast-synthesis model 3

ASM1 = arm-synthesis model 1

ASM2 = arm-synthesis model2

ratio = ratio of the number of treatments to the number of studies

β_0 = constant (i.e., $ln(OR)$ of contrast-synthesis model 1 (CSM1) when ratio = 0)

$\beta_1, \beta_2, \beta_3, \beta_4$ represent the expected differences between each of the models CSM2, CSM3, ASM1, ASM2 and CSM1, respectively when the ratio is 0

$\beta_5 =$ Slope between the ratio and the effect estimate for CSM1

$\beta_6, \beta_7, \beta_8, \beta_9$ represent the expected differences between CSM2, CSM3, ASM1, ASM2 and CSM1 when ratio is 0

u_i represents the random-effect for network

$v_{j(i)}$ represents the random-effect for treatment within network

e_{ij} represents the random error, and $var(e_{ij}) = \sigma_k^2$; where $k = model$

First, we calculate the ratio of the odds ratio (OR) at the value of the ratio of the 'number of treatments to the number of studies' at the 75th percentile (R_{75}) and 25th percentile (R_{25}) for contrast-synthesis model 1 (i.e., what is the difference at R_{75} removing out any systematic differences between the models at R_{25}?).

1) Ratio of ORs calculated at R_{75} and R_{25} for contrast-synthesis model 1:

$$\exp(\ln(OR)_{CSM_1,R_{75}} - \ln(OR)_{CSM_1,R_{25}})$$
$$= \exp((\beta_0 + \beta_5 \times R_{75}) - (\beta_0 + \beta_5 \times R_{25}))$$
$$= \exp((\beta_5 \times R_{75} - \beta_5 \times R_{25}))$$
$$= \exp(\beta_5 \times (R_{75} - R_{25})) \quad \textit{Equation 1}$$

2) Ratio of ORs calculated at R_{75} and R_{25} for contrast-synthesis model 2 is provided in Equation 2 below. To calculate the ratio for contrast-synthesis model 3, arm-synthesis model 1 and arm-synthesis model 2, Equation 2 is used but β_6 is substituted with β_7, β_8, and β_9, respectively.

$$exp(ln(OR)_{CSM_2,R_{75}} - ln(OR)_{CSM_2,R_{25}})$$
$$= exp((\beta_0 + \beta_1 + \beta_5 \times R_{75} + \beta_6 \times R_{75}) - (\beta_0 + \beta_1 + \beta_5 \times R_{25} + \beta_6 \times R_{25}))$$
$$= exp((\beta_5 \times R_{75} + \beta_6 \times R_{75}) - (\beta_5 \times R_{25} + \beta_6 \times R_{25}))$$
$$= \exp(\beta_5 \times (R_{75} - R_{25}) + \beta_6 \times (R_{75} - R_{25})) \quad \text{Equation 2}$$

Next, we compare the ratio of the odds ratio calculated for each model above with the ratio of odds ratios calculated for contrast-synthesis model 1 (i.e., the reference model). We show this for the comparison of contrast-synthesis model 2 to contrast-synthesis model 1 but this can easily be extended for comparison of the other synthesis models to contrast-synthesis model 1.

Comparing CSM2 to CSM1:

$$\text{Equation } 2 \div \textit{Equation } 1$$
$$= \exp(ln(OR)_{CSM_2,R_{75}} - ln(OR)_{CSM_2,R_{25}}) \div \exp(\ln(OR)_{CSM_1,R_{75}} - \ln(OR)_{CSM_1,R_{25}})$$
$$= \exp(\beta_5 \times (R_{75} - R_{25}) + \beta_6 \times (R_{75} - R_{25})) \div \exp(\beta_5 \times (R_{75} - R_{25}))$$
$$= \exp\big(\beta_6 * (R_{75} - R_{25})\big) \quad \textit{Equation 3}$$

Supplementary Results

Supplementary Table S2. Characteristics of the included networks

Network ID	Number of treatments	Number of studies	Number of comparisons (direct and indirect)	Ratio of treatments to studies	Ratio of treatments to unique direct comparisons	Ratio of studies to unique direct comparisons	Proportion of treatment arms with <10 events[a]	Comparison type[b]	Outcome type (Objective/ semi-objective/ subjective)
**473552**	19	32	171	0.59	0.73	1.23	0.36	pharmacological vs placebo	Semi-objective
**476033**	5	7	10	0.71	1.25	1.75	0.00	pharmacological vs placebo	Objective
**479585**	10	49	45	0.20	1.11	5.44	0.19	pharmacological vs placebo	Semi-objective
**479603**	45	83	990	0.54	0.75	1.38	0.40	pharmacological vs placebo	Semi-objective
**479650**	7	28	21	0.25	0.64	2.55	0.00	pharmacological vs placebo	Objective
**479661**	4	14	6	0.29	0.80	2.80	0.00	pharmacological vs pharmacological	Objective
**479664**	13	95	78	0.14	0.39	2.88	0.14	non-pharmacological vs any	Semi-objective
**479770**	5	46	10	0.11	0.63	5.75	0.08	pharmacological vs placebo	Objective
**479773**	5	11	10	0.45	1.25	2.75	0.00	pharmacological vs placebo	Objective
**479808**	7	31	21	0.23	1.17	5.17	0.28	pharmacological vs placebo	Semi-objective

Continued…

Network ID	Number of treatments	Number of studies	Number of comparisons (direct and indirect)	Ratio of treatments to studies	Ratio of treatments to unique direct comparisons	Ratio of studies to unique direct comparisons	Proportion of treatment arms with <10 events[a]	Comparison type[b]	Outcome type (Objective/ semi-objective/ subjective)
**479918**	7	15	21	0.47	1.17	2.50	0.03	pharmacological vs placebo	Objective
**479969**	21	95	210	0.22	0.62	2.79	0.18	non-pharmacological vs any	Semi-objective
**479971**	4	27	6	0.15	1.33	9.00	0.00	pharmacological vs placebo	Objective
**479996**	9	21	36	0.43	0.82	1.91	0.20	pharmacological vs placebo	Semi-objective
**480029**	8	17	28	0.47	0.89	1.89	0.00	pharmacological vs pharmacological	Objective
**480037**	7	11	21	0.64	1.17	1.83	0.18	pharmacological vs placebo	Semi-objective
**480052**	8	20	28	0.40	0.73	1.82	0.14	pharmacological vs placebo	Semi-objective
**480060**	5	46	10	0.11	0.71	6.57	0.00	pharmacological vs placebo	Objective
**480074**	5	16	10	0.31	1.25	4.00	0.50	non-pharmacological vs any	Objective
**480629**	10	23	45	0.43	1.00	2.30	0.23	pharmacological vs placebo	Semi-objective
**480666**	29	45	406	0.64	0.67	1.05	0.31	pharmacological vs placebo	Subjective

Continued…

Network ID	Number of treatments	Number of studies	Number of comparisons (direct and indirect)	Ratio of treatments to studies	Ratio of treatments to unique direct comparisons	Ratio of studies to unique direct comparisons	Proportion of treatment arms with <10 events[a]	Comparison type[b]	Outcome type (Objective/ semi-objective/ subjective)
**480706**	5	8	10	0.63	1.00	1.60	0.11	non-pharmacological vs any	Semi-objective
**480804**	6	19	15	0.32	0.86	2.71	0.66	pharmacological vs placebo	Objective
**480931**	6	13	15	0.46	0.86	1.86	0.30	pharmacological vs pharmacological	Objective
**480935**	5	8	10	0.63	1.00	1.60	0.00	pharmacological vs placebo	Semi-objective
**480967**	26	60	325	0.43	0.36	0.82	0.01	pharmacological vs placebo	Subjective
**481147**	4	5	6	0.80	1.33	1.67	0.10	pharmacological vs placebo	Objective
**481150**	11	40	55	0.28	0.61	2.22	0.00	pharmacological vs pharmacological	Objective
**481159**	5	8	10	0.63	1.25	2.00	0.06	pharmacological vs placebo	Objective
**481163**	8	10	28	0.80	1.14	1.43	0.20	pharmacological vs placebo	Semi-objective
**481193**	14	45	91	0.31	0.50	1.61	0.13	pharmacological vs placebo	Subjective

Continued…

Network ID	Number of treatments	Number of studies	Number of comparisons (direct and indirect)	Ratio of treatments to studies	Ratio of treatments to unique direct comparisons	Ratio of studies to unique direct comparisons	Proportion of treatment arms with <10 events[a]	Comparison type[b]	Outcome type (Objective/ semi-objective/ subjective)
**481216**	5	18	10	0.28	1.00	3.60	0.19	pharmacological vs placebo	Semi-objective
**481236**	4	6	6	0.67	1.33	2.00	0.33	pharmacological vs placebo	Semi-objective
**481323**	9	13	36	0.69	0.60	0.87	0.00	non-pharmacological vs any	Objective
**481378**	13	94	78	0.14	0.38	2.76	0.06	pharmacological vs placebo	Objective
**481551**	7	14	21	0.50	0.88	1.75	0.00	pharmacological vs pharmacological	Objective
**481588**	18	27	153	0.74	1.00	1.35	0.05	pharmacological vs placebo	Semi-objective
**481589**	10	41	45	0.24	1.11	4.56	0.00	pharmacological vs placebo	Objective
**481731**	6	14	15	0.43	1.00	2.33	0.00	pharmacological vs placebo	Objective
**481733**	4	12	6	0.33	0.67	2.00	0.23	pharmacological vs placebo	Objective
**481734**	6	28	15	0.21	0.43	2.00	0.00	pharmacological vs placebo	Semi-objective
**481766**	7	8	21	0.88	0.78	0.89	0.00	pharmacological vs placebo	Semi-objective

Continued…

Network ID	Number of treatments	Number of studies	Number of comparisons (direct and indirect)	Ratio of treatments to studies	Ratio of treatments to unique direct comparisons	Ratio of studies to unique direct comparisons	Proportion of treatment arms with <10 events[a]	Comparison type[b]	Outcome type (Objective/ semi-objective/ subjective)
**481942**	6	41	15	0.15	0.67	4.56	0.40	pharmacological vs pharmacological	Semi-objective
**482001**	7	102	21	0.07	0.88	12.75	0.29	non-pharmacological vs any	Subjective
**482004**	8	25	28	0.32	1.00	3.13	0.27	pharmacological vs placebo	Semi-objective
**482120**	9	18	36	0.50	1.13	2.25	0.00	pharmacological vs placebo	Objective
**482159**	6	30	15	0.20	0.46	2.31	0.10	pharmacological vs placebo	Semi-objective
**482416**	7	8	21	0.88	1.17	1.33	0.00	pharmacological vs placebo	Objective
**482440**	5	16	10	0.31	0.83	2.67	0.00	pharmacological vs placebo	Semi-objective
**482465**	10	15	45	0.67	0.91	1.36	0.09	pharmacological vs placebo	Semi-objective
**482476**	9	15	36	0.60	0.90	1.50	0.52	non-pharmacological vs any	Objective
**482519**	7	11	21	0.64	0.58	0.92	0.33	pharmacological vs placebo	Objective
**482521**	4	6	6	0.67	1.33	2.00	0.25	pharmacological vs placebo	Semi-objective

Continued…

Network ID	Number of treatments	Number of studies	Number of comparisons (direct and indirect)	Ratio of treatments to studies	Ratio of treatments to unique direct comparisons	Ratio of studies to unique direct comparisons	Proportion of treatment arms with <10 events[a]	Comparison type[b]	Outcome type (Objective/ semi-objective/ subjective)
**482522**	4	6	6	0.67	1.33	2.00	0.17	pharmacological vs placebo	Semi-objective
**482576**	5	11	10	0.45	1.00	2.20	0.18	pharmacological vs placebo	Semi-objective
**482734**	4	48	6	0.08	0.80	9.60	0.66	non-pharmacological vs any	Semi-objective
**501192**	8	18	28	0.44	1.14	2.57	0.50	pharmacological vs placebo	Subjective
**501194**	17	99	136	0.17	0.74	4.30	0.02	pharmacological vs placebo	Semi-objective
**501197**	5	11	10	0.45	0.63	1.38	0.00	pharmacological vs placebo	Objective
**501201**	8	38	28	0.21	0.50	2.38	0.03	pharmacological vs placebo	Subjective
**501206**	8	49	28	0.16	0.44	2.72	0.00	pharmacological vs placebo	Objective
**501207**	8	22	28	0.36	0.89	2.44	0.38	pharmacological vs placebo	Subjective
**501212**	5	6	10	0.83	1.25	1.50	0.00	pharmacological vs pharmacological	Objective
**501215**	7	10	21	0.70	0.78	1.11	0.00	pharmacological vs placebo	Subjective

Continued…

Network ID	Number of treatments	Number of studies	Number of comparisons (direct and indirect)	Ratio of treatments to studies	Ratio of treatments to unique direct comparisons	Ratio of studies to unique direct comparisons	Proportion of treatment arms with <10 events[a]	Comparison type[b]	Outcome type (Objective/ semi-objective/ subjective)
**501217**	6	38	15	0.16	0.60	3.80	0.00	pharmacological vs placebo	Semi-objective
**501224**	9	11	36	0.82	0.82	1.00	0.00	pharmacological vs placebo	Objective
**501226**	5	12	10	0.42	1.00	2.40	0.25	non-pharmacological vs any	Subjective
**501227**	8	21	28	0.38	0.89	2.33	0.00	pharmacological vs placebo	Objective
**501228**	9	46	36	0.20	1.00	5.11	0.17	non-pharmacological vs any	Semi-objective
**501235**	7	20	21	0.35	0.70	2.00	0.15	non-pharmacological vs any	Semi-objective
**501250**	8	20	28	0.40	0.73	1.82	0.00	pharmacological vs placebo	Objective
**501251**	6	41	15	0.15	0.60	4.10	0.00	pharmacological vs placebo	Objective
**501253**	11	13	55	0.85	0.58	0.68	0.21	non-pharmacological vs any	Subjective
**501256**	5	61	10	0.08	1.00	12.20	0.26	pharmacological vs placebo	Semi-objective

Continued…

Network ID	Number of treatments	Number of studies	Number of comparisons (direct and indirect)	Ratio of treatments to studies	Ratio of treatments to unique direct comparisons	Ratio of studies to unique direct comparisons	Proportion of treatment arms with <10 events[a]	Comparison type[b]	Outcome type (Objective/ semi-objective/ subjective)
**501257**	6	22	15	0.27	0.43	1.57	0.00	pharmacological vs placebo	Semi-objective
**501261**	10	21	45	0.48	0.77	1.62	0.29	pharmacological vs placebo	Semi-objective
**501267**	9	26	36	0.35	0.56	1.63	0.00	pharmacological vs placebo	Subjective
**501268**	5	37	10	0.14	1.25	9.25	0.04	pharmacological vs placebo	Objective
**501272**	14	64	91	0.22	0.39	1.78	0.01	pharmacological vs placebo	Subjective
**501283**	17	55	136	0.31	0.59	1.90	0.06	pharmacological vs placebo	Semi-objective
**501287**	7	14	21	0.50	0.44	0.88	0.05	pharmacological vs placebo	Semi-objective
**501297**	4	82	6	0.05	0.67	13.67	0.02	pharmacological vs placebo	Objective
**501302**	6	8	15	0.75	1.20	1.60	0.00	pharmacological vs placebo	Semi-objective
**501305**	5	12	10	0.42	1.25	3.00	0.00	pharmacological vs placebo	Semi-objective
**501311**	5	14	10	0.36	1.25	3.50	0.00	pharmacological vs placebo	Semi-objective
**501313**	8	19	28	0.42	1.14	2.71	0.05	pharmacological vs placebo	Subjective

Continued…

Network ID	Number of treatments	Number of studies	Number of comparisons (direct and indirect)	Ratio of treatments to studies	Ratio of treatments to unique direct comparisons	Ratio of studies to unique direct comparisons	Proportion of treatment arms with <10 events[a]	Comparison type[b]	Outcome type (Objective/ semi-objective/ subjective)
**501318**	7	17	21	0.41	0.88	2.13	0.37	pharmacological vs placebo	Semi-objective
**501325**	5	26	10	0.19	1.25	6.50	0.00	pharmacological vs placebo	Subjective
**501330**	7	29	21	0.24	1.17	4.83	0.17	pharmacological vs placebo	Subjective
**501336**	4	8	6	0.50	1.33	2.67	0.19	pharmacological vs placebo	Subjective
**501345**	5	19	10	0.26	1.25	4.75	0.00	pharmacological vs placebo	Objective
**501346**	7	58	21	0.12	1.17	9.67	0.00	pharmacological vs placebo	Objective
**501348**	4	89	6	0.04	0.80	17.80	0.15	pharmacological vs placebo	Semi-objective
**501350**	11	40	55	0.28	0.61	2.22	0.46	pharmacological vs placebo	Semi-objective
**501351**	8	101	28	0.08	0.36	4.59	0.00	pharmacological vs placebo	Semi-objective
**501367**	6	20	15	0.30	0.75	2.50	0.00	pharmacological vs placebo	Semi-objective
**501370**	5	10	10	0.50	1.00	2.00	0.00	pharmacological vs placebo	Objective
**501371**	4	7	6	0.57	1.33	2.33	0.00	pharmacological vs placebo	Objective

Continued…

Network ID	Number of treatments	Number of studies	Number of comparisons (direct and indirect)	Ratio of treatments to studies	Ratio of treatments to unique direct comparisons	Ratio of studies to unique direct comparisons	Proportion of treatment arms with <10 events[a]	Comparison type[b]	Outcome type (Objective/ semi-objective/ subjective)
**501373**	7	28	21	0.25	0.58	2.33	0.00	pharmacological vs placebo	Objective
**501374**	5	34	10	0.15	0.56	3.78	0.00	pharmacological vs placebo	Subjective
**501377**	8	17	28	0.47	0.73	1.55	0.44	pharmacological vs placebo	Subjective
**501379**	4	39	6	0.10	0.80	7.80	0.43	pharmacological vs placebo	Subjective
**501381**	10	12	45	0.83	0.83	1.00	0.17	pharmacological vs placebo	Subjective
**501382**	11	17	55	0.65	0.73	1.13	0.00	pharmacological vs placebo	Semi-objective
**501384**	15	60	105	0.25	0.50	2.00	0.18	pharmacological vs placebo	Semi-objective
**501388**	14	34	91	0.41	0.70	1.70	0.25	pharmacological vs placebo	Semi-objective
**501392**	12	51	66	0.24	0.52	2.22	0.07	pharmacological vs placebo	Objective
**501393**	7	27	21	0.26	1.17	4.50	0.28	pharmacological vs placebo	Subjective
**501395**	8	14	28	0.57	0.73	1.27	0.22	pharmacological vs placebo	Semi-objective
**501404**	5	23	10	0.22	0.71	3.29	0.00	pharmacological vs placebo	Semi-objective

Continued…

Network ID	Number of treatments	Number of studies	Number of comparisons (direct and indirect)	Ratio of treatments to studies	Ratio of treatments to unique direct comparisons	Ratio of studies to unique direct comparisons	Proportion of treatment arms with <10 events[a]	Comparison type[b]	Outcome type (Objective/ semi-objective/ subjective)
**501412**	6	14	15	0.43	1.20	2.80	0.21	pharmacological vs placebo	Subjective
**501418**	4	9	6	0.44	1.00	2.25	0.00	pharmacological vs placebo	Semi-objective
**501420**	5	10	10	0.50	1.00	2.00	0.00	pharmacological vs placebo	Subjective
**501424**	9	43	36	0.21	0.75	3.58	0.02	non-pharmacological vs any	Semi-objective
**501425**	16	36	120	0.44	0.84	1.89	0.00	non-pharmacological vs any	Objective
**501430**	10	19	45	0.53	0.83	1.58	0.22	pharmacological vs placebo	Objective
**501431**	11	62	55	0.18	0.55	3.10	0.07	pharmacological vs placebo	Objective
**501434**	6	14	15	0.43	0.67	1.56	0.00	pharmacological vs placebo	Semi-objective

[a] Ratio of rare studies = the number of treatment arms with fewer than 10 events divided by the total number of comparisons within the network.
[b] Each network is categorised according to the type of its included treatment comparisons and outcomes. Specifically, in the presence of placebo in the network, the network was categorized as pharmacological vs. placebo. If only pharmacological treatments were available then we categorized the network as pharmacological vs pharmacological, whereas if a non-pharmacological treatment was included in the network, then we selected non-pharmacological vs any category.

Continued…

Supplementary Table S3. Summary characteristics of networks

Factor	Level	Median (IQR) or n (%) (N = 118)
Number of treatments		7.0 (5.0, 9.0)
Number of studies		20.0 (13.0, 40.0)
Comparison type	Non-pharmacological vs any	13 (11.0%)
	Pharmacological vs pharmacological	4 (3.4%)
	Pharmacological vs placebo	101 (85.6%)
Outcome type	Harmful	61 (51.7%)
	Beneficial	57 (48.3%)
Outcome type	Objective	43 (36.4%)
	Semi-objective	52 (44.1%)
	Subjective	23 (19.5%)
Ratio of treatments to studies		0.4 (0.2, 0.6)
Ratio of treatments to comparisons		0.9 (0.7, 1.1)
Ratio of studies to unique direct comparisons		2.3 (1.6, 3.6)
Proportion of arms with <10 events		0.02 (0.00, 0.19)

Abbreviations: IQR, inter-quartile range.

Convergence and estimation

After examination of the convergence diagnostics, we found no issues with convergence for contrast-synthesis model 2 or arm-synthesis model 1. Five networks were determined to have issues with convergence when fitting contrast-synthesis model 1. We re-fitted these networks and saved every 500th sample instead of every 10th, which resolved the convergence issues. For arm-synthesis model 2, 30 networks had issues with convergence. These issues resolved for 5 networks after re-running the models and saving every 500th sample; leaving 25 networks with persistent issues in their convergence diagnostics which were excluded from arm-synthesis model 2 (Supplementary Table S4 and Online material https://nma.emilykarahalios.com/).

Of the 118 included networks, two networks failed to yield one or more estimates (i.e., $ln(OR)$, or the $SE(ln(OR))$) using arm-synthesis models 1 and 2, and another network failed to yield estimates using arm-synthesis model 2 (Details provided in Supplementary Table S5). All networks yielded estimates for the contrast-synthesis models.

Supplementary Table S4. Characteristics of networks and treatments that did not appear to converge for arm-synthesis model 2 after inspection of the convergence diagnostics.

**Network**	**Treatment #**	**Number of studies**	**Number of possible (direct and indirect) comparisons**	**Mean proportion of events**	**Description of convergence issue using arm-synthesis model 2**
473552	1	2	171	0.31 (0.28, 0.33)	Trace/density plots do not show convergence
473552	2	24	171	0.05 (0.00, 0.28)	
473552	3	3	171	0.17 (0.09, 0.21)	
473552	4	3	171	0.11 (0.11, 0.14)	
473552	5	2	171	0.19 (0.17, 0.21)	
473552	6	8	171	0.02 (0.00, 0.20)	
473552	7	2	171	0.13 (0.12, 0.15)	
473552	8	1	171	0.34 (0.34, 0.34)	
473552	9	4	171	0.14 (0.12, 0.26)	
473552	10	3	171	0.20 (0.19, 0.46)	
473552	11	3	171	0.36 (0.15, 0.48)	
473552	12	3	171	0.14 (0.12, 0.17)	
473552	13	2	171	0.15 (0.13, 0.16)	
473552	14	1	171	0.05 (0.05, 0.05)	
473552	15	3	171	0.14 (0.10, 0.21)	
473552	16	1	171	0.07 (0.07, 0.07)	
473552	17	3	171	0.15 (0.12, 0.17)	
473552	18	1	171	0.23 (0.23, 0.23)	
473552	19	1	171	0.15 (0.15, 0.15)	
479770	1	36	10	1.00 (0.68, 1.00)	Trace/density plots do not show convergence
479770	2	26	10	0.98 (0.75, 1.00)	
479770	3	3	10	1.00 (1.00, 1.00)	

Network	Treatment #	Number of studies	Number of possible (direct and indirect) comparisons	Mean proportion of events	Description of convergence issue using arm-synthesis model 2
479770	4	16	10	0.99 (0.80, 1.00)	
479770	5	11	10	1.00 (0.70, 1.00)	
479773	1	11	10	0.90 (0.82, 0.96)	Trace/density plots do not show convergence
479773	2	5	10	0.90 (0.88, 0.98)	
479773	3	1	10	0.91 (0.91, 0.91)	
479773	4	4	10	0.94 (0.91, 0.96)	
479773	5	1	10	0.95 (0.95, 0.95)	
479918	1	15	21	0.18 (0.10, 0.28)	Trace/density plots do not show convergence
479918	2	4	21	0.34 (0.31, 0.45)	
479918	3	3	21	0.35 (0.29, 0.44)	
479918	4	3	21	0.26 (0.20, 0.32)	
479918	5	3	21	0.38 (0.37, 0.39)	
479918	6	1	21	0.43 (0.43, 0.43)	
479918	7	1	21	0.33 (0.33, 0.33)	
480629	1	23	45	0.10 (0.03, 0.40)	Trace/density plots do not show convergence
480629	2	2	45	0.35 (0.30, 0.40)	
480629	3	4	45	0.35 (0.24, 0.43)	
480629	4	3	45	0.17 (0.16, 0.17)	
480629	5	3	45	0.33 (0.23, 0.33)	
480629	6	4	45	0.37 (0.32, 0.50)	
480629	7	1	45	0.29 (0.29, 0.29)	
480629	8	2	45	0.33 (0.32, 0.34)	
480629	9	1	45	0.39 (0.39, 0.39)	
480629	10	4	45	0.41 (0.37, 0.49)	
480666	1	23	406	0.29 (0.00, 0.47)	Trace/density plots do not show convergence
480666	2	10	406	0.48 (0.14, 0.63)	
480666	3	8	406	0.53 (0.19, 0.76)	
480666	4	4	406	0.55 (0.49, 0.67)	
480666	5	1	406	0.65 (0.65, 0.65)	
480666	6	1	406	0.40 (0.40, 0.40)	
480666	7	2	406	0.70 (0.69, 0.71)	
480666	8	1	406	0.68 (0.68, 0.68)	
480666	9	1	406	0.62 (0.62, 0.62)	
480666	10	13	406	0.55 (0.34, 0.94)	
480666	11	1	406	0.62 (0.62, 0.62)	
480666	12	1	406	0.47 (0.47, 0.47)	
480666	13	1	406	0.22 (0.22, 0.22)	
480666	14	1	406	0.28 (0.28, 0.28)	

Network	Treatment #	Number of studies	Number of possible (direct and indirect) comparisons	Mean proportion of events	Description of convergence issue using arm-synthesis model 2
480666	15	1	406	0.45 (0.45, 0.45)	
480666	16	1	406	0.44 (0.44, 0.44)	
480666	17	4	406	0.50 (0.33, 0.88)	
480666	18	10	406	0.52 (0.43, 0.83)	
480666	19	1	406	0.43 (0.43, 0.43)	
480666	20	1	406	0.15 (0.15, 0.15)	
480666	21	2	406	0.25 (0.10, 0.40)	
480666	22	2	406	0.39 (0.33, 0.44)	
480666	23	1	406	0.74 (0.74, 0.74)	
480666	24	1	406	0.56 (0.56, 0.56)	
480666	25	2	406	0.45 (0.41, 0.49)	
480666	26	1	406	0.18 (0.18, 0.18)	
480666	27	1	406	0.66 (0.66, 0.66)	
480666	28	1	406	0.46 (0.46, 0.46)	
480666	29	1	406	0.50 (0.50, 0.50)	
480931	1	8	15	0.61 (0.25, 0.76)	Trace/density plots do not show convergence
480931	2	3	15	0.50 (0.29, 0.69)	
480931	3	6	15	0.55 (0.50, 0.59)	
480931	4	1	15	0.55 (0.55, 0.55)	
480931	5	3	15	0.57 (0.40, 0.66)	
480931	6	6	15	0.65 (0.49, 0.75)	
480967	1	33	325	0.96 (0.79, 1.00)	Trace plots do not show convergence
480967	2	27	325	0.91 (0.76, 0.99)	
480967	3	11	325	0.97 (0.88, 1.00)	
480967	4	6	325	0.89 (0.88, 1.00)	
480967	5	12	325	0.88 (0.80, 1.00)	
480967	6	4	325	0.94 (0.90, 0.98)	
480967	7	9	325	0.89 (0.82, 0.94)	
480967	8	5	325	0.96 (0.93, 1.00)	
480967	9	2	325	0.88 (0.87, 0.88)	
480967	10	5	325	0.94 (0.91, 1.00)	
480967	11	9	325	0.93 (0.88, 0.97)	
480967	12	3	325	0.88 (0.87, 0.91)	
480967	13	5	325	0.89 (0.82, 0.92)	
480967	14	1	325	0.82 (0.82, 0.82)	
480967	15	7	325	0.90 (0.87, 0.91)	
480967	16	1	325	0.79 (0.79, 0.79)	
480967	17	1	325	0.88 (0.88, 0.88)	
480967	18	1	325	0.74 (0.74, 0.74)	

Network	Treatment #	Number of studies	Number of possible (direct and indirect) comparisons	Mean proportion of events	Description of convergence issue using arm-synthesis model 2
480967	19	1	325	0.92 (0.92, 0.92)	
480967	20	1	325	0.78 (0.78, 0.78)	
480967	21	2	325	0.91 (0.83, 1.00)	
480967	22	1	325	0.82 (0.82, 0.82)	
480967	23	1	325	0.83 (0.83, 0.83)	
480967	24	1	325	0.92 (0.92, 0.92)	
480967	25	1	325	0.94 (0.94, 0.94)	
480967	26	1	325	0.86 (0.86, 0.86)	
481551	1	12	21	0.96 (0.85, 1.00)	Bimodal density distributions obtained
481551	2	6	21	0.99 (0.94, 1.00)	
481551	3	5	21	0.94 (0.93, 1.00)	
481551	4	1	21	0.93 (0.93, 0.93)	
481551	5	1	21	1.00 (1.00, 1.00)	
481551	6	3	21	0.98 (0.92, 1.00)	
481551	7	1	21	1.00 (1.00, 1.00)	
481589	1	5	45	0.89 (0.78, 0.94)	Trace/density plots do not show convergence
481589	2	41	45	0.81 (0.13, 0.96)	
481589	3	7	45	0.82 (0.76, 0.91)	
481589	4	4	45	0.78 (0.76, 0.88)	
481589	5	3	45	0.70 (0.68, 0.74)	
481589	6	6	45	0.83 (0.76, 0.97)	
481589	7	3	45	0.95 (0.89, 0.98)	
481589	8	5	45	0.90 (0.79, 0.92)	
481589	9	5	45	0.91 (0.82, 0.98)	
481589	10	3	45	0.88 (0.86, 0.93)	
482004	1	25	28	0.22 (0.00, 0.55)	Trace/density plots do not show convergence
482004	2	3	28	0.43 (0.41, 0.47)	
482004	3	10	28	0.47 (0.22, 0.71)	
482004	4	4	28	0.47 (0.41, 0.52)	
482004	5	3	28	0.53 (0.46, 0.56)	
482004	6	2	28	0.34 (0.29, 0.39)	
482004	7	3	28	0.65 (0.27, 0.78)	
482004	8	2	28	0.28 (0.27, 0.30)	
482120	1	18	36	0.99 (0.91, 1.00)	Trace/density plots do not show convergence
482120	2	6	36	1.00 (0.99, 1.00)	
482120	3	1	36	0.99 (0.99, 0.99)	
482120	4	3	36	0.98 (0.97, 0.99)	
482120	5	1	36	0.99 (0.99, 0.99)	

Network	Treatment #	Number of studies	Number of possible (direct and indirect) comparisons	Mean proportion of events	Description of convergence issue using arm-synthesis model 2
482120	6	4	36	0.98 (0.98, 1.00)	
482120	7	1	36	0.96 (0.96, 0.96)	
482120	8	1	36	1.00 (1.00, 1.00)	
482120	9	1	36	0.99 (0.99, 0.99)	
482416	1	8	21	0.84 (0.61, 0.98)	Bimodal density distributions obtained
482416	2	1	21	0.89 (0.89, 0.89)	
482416	3	1	21	0.93 (0.93, 0.93)	
482416	4	3	21	0.85 (0.80, 0.91)	
482416	5	1	21	0.90 (0.90, 0.90)	
482416	6	1	21	0.96 (0.96, 0.96)	
482416	7	1	21	1.00 (1.00, 1.00)	
501194	1	95	136	0.90 (0.25, 1.00)	Trace/density plots do not show convergence
501194	2	6	136	0.89 (0.45, 0.98)	
501194	3	4	136	0.94 (0.90, 0.97)	
501194	4	18	136	0.96 (0.75, 1.00)	
501194	5	10	136	0.96 (0.92, 1.00)	
501194	6	22	136	0.95 (0.65, 1.00)	
501194	7	3	136	0.89 (0.87, 0.96)	
501194	8	3	136	0.92 (0.92, 0.98)	
501194	9	9	136	0.93 (0.90, 0.98)	
501194	10	6	136	0.94 (0.88, 0.98)	
501194	11	8	136	0.95 (0.92, 1.00)	
501194	12	10	136	0.94 (0.84, 0.97)	
501194	13	3	136	0.89 (0.81, 0.91)	
501194	14	2	136	0.96 (0.91, 1.00)	
501194	15	2	136	0.90 (0.88, 0.93)	
501194	16	3	136	0.95 (0.94, 0.97)	
501194	17	2	136	0.99 (0.98, 1.00)	
501197	1	3	10	0.95 (0.95, 0.98)	Bimodal density distributions obtained
501197	2	6	10	0.98 (0.95, 0.99)	
501197	3	7	10	0.96 (0.91, 1.00)	
501197	4	5	10	0.97 (0.95, 0.99)	
501197	5	3	10	0.98 (0.98, 1.00)	
501250	1	8	28	0.94 (0.76, 0.97)	Trace/density plots do not show convergence
501250	2	16	28	0.97 (0.91, 1.00)	
501250	3	13	28	0.95 (0.78, 1.00)	
501250	4	2	28	0.95 (0.92, 0.97)	
501250	5	1	28	0.97 (0.97, 0.97)	

Network	Treatment #	Number of studies	Number of possible (direct and indirect) comparisons	Mean proportion of events	Description of convergence issue using arm-synthesis model 2
501250	6	1	28	0.98 (0.98, 0.98)	
501250	7	1	28	0.97 (0.97, 0.97)	
501250	8	2	28	0.98 (0.98, 0.98)	
501251	1	33	15	0.98 (0.85, 1.00)	Trace/density plots do not show convergence
501251	2	2	15	0.97 (0.97, 0.97)	
501251	3	12	15	0.98 (0.87, 1.00)	
501251	4	15	15	0.98 (0.84, 1.00)	
501251	5	17	15	0.99 (0.88, 1.00)	
501251	6	20	15	0.99 (0.87, 1.00)	
501267	1	11	36	0.90 (0.65, 0.98)	Trace/density plots do not show convergence
501267	2	1	36	1.00 (1.00, 1.00)	
501267	3	14	36	0.93 (0.68, 0.99)	
501267	4	4	36	0.88 (0.85, 0.97)	
501267	5	5	36	0.94 (0.83, 0.97)	
501267	6	5	36	0.96 (0.83, 0.97)	
501267	7	11	36	0.95 (0.84, 0.99)	
501267	8	2	36	0.95 (0.94, 0.95)	
501267	9	4	36	0.94 (0.89, 1.00)	
501283	1	11	136	0.56 (0.09, 0.96)	Trace plots do not show convergence
501283	2	7	136	0.91 (0.81, 0.97)	
501283	3	1	136	0.81 (0.81, 0.81)	
501283	4	18	136	0.85 (0.38, 0.96)	
501283	5	20	136	0.83 (0.39, 0.96)	
501283	6	21	136	0.81 (0.44, 0.98)	
501283	7	11	136	0.85 (0.45, 0.95)	
501283	8	2	136	0.90 (0.88, 0.93)	
501283	9	4	136	0.89 (0.75, 0.95)	
501283	10	10	136	0.84 (0.65, 0.93)	
501283	11	1	136	0.76 (0.76, 0.76)	
501283	12	1	136	0.94 (0.94, 0.94)	
501283	13	1	136	0.73 (0.73, 0.73)	
501283	14	1	136	0.70 (0.70, 0.70)	
501283	15	1	136	0.85 (0.85, 0.85)	
501283	16	1	136	0.90 (0.90, 0.90)	
501283	17	1	136	0.55 (0.55, 0.55)	
501350	1	18	55	0.03 (0.00, 0.11)	Trace/density plots do not show convergence
501350	2	1	55	0.00 (0.00, 0.00)	
501350	3	1	55	0.01 (0.01, 0.01)	

Network	Treatment #	Number of studies	Number of possible (direct and indirect) comparisons	Mean proportion of events	Description of convergence issue using arm-synthesis model 2
501350	4	4	55	0.01 (0.00, 0.02)	
501350	5	2	55	0.02 (0.01, 0.02)	
501350	6	5	55	0.01 (0.01, 0.02)	
501350	7	1	55	0.01 (0.01, 0.01)	
501350	8	10	55	0.01 (0.00, 0.03)	
501350	9	10	55	0.03 (0.00, 0.10)	
501350	10	28	55	0.02 (0.00, 0.08)	
501350	11	5	55	0.02 (0.01, 0.07)	
501367	1	19	15	0.86 (0.53, 0.98)	Trace/density plots do not show convergence
501367	2	2	15	0.90 (0.89, 0.90)	
501367	3	8	15	0.89 (0.60, 1.00)	
501367	4	7	15	0.94 (0.68, 1.00)	
501367	5	5	15	0.97 (0.89, 1.00)	
501367	6	1	15	0.64 (0.64, 0.64)	
501382	1	5	55	0.93 (0.82, 0.95)	Trace/density plots do not show convergence
501382	2	4	55	0.95 (0.91, 0.97)	
501382	3	2	55	0.96 (0.94, 0.97)	
501382	4	1	55	0.99 (0.99, 0.99)	
501382	5	2	55	0.94 (0.92, 0.95)	
501382	6	2	55	0.97 (0.97, 0.97)	
501382	7	1	55	0.97 (0.97, 0.97)	
501382	8	1	55	0.98 (0.98, 0.98)	
501382	9	16	55	0.98 (0.93, 1.00)	
501382	10	1	55	0.96 (0.96, 0.96)	
501382	11	2	55	0.98 (0.98, 0.98)	
501392	1	8	66	0.41 (0.19, 0.73)	Trace/density plots do not show convergence
501392	2	18	66	0.58 (0.24, 0.97)	
501392	3	14	66	0.66 (0.48, 0.93)	
501392	4	25	66	0.50 (0.25, 0.96)	
501392	5	17	66	0.83 (0.46, 0.97)	
501392	7	2	66	0.83 (0.79, 0.86)	
501392	8	2	66	0.70 (0.67, 0.73)	
501392	9	7	66	0.69 (0.49, 0.93)	
501392	10	3	66	0.92 (0.67, 1.00)	
501392	11	2	66	0.99 (0.98, 1.00)	
501392	12	6	66	0.57 (0.20, 0.74)	
501425	1	36	120	0.96 (0.78, 1.00)	Trace/density plots do not show convergence
501425	2	3	120	0.95 (0.94, 0.97)	

Network	Treatment #	Number of studies	Number of possible (direct and indirect) comparisons	Mean proportion of events	Description of convergence issue using arm-synthesis model 2
501425	3	6	120	0.97 (0.95, 1.00)	
501425	4	7	120	0.97 (0.82, 1.00)	
501425	5	1	120	0.99 (0.99, 0.99)	
501425	6	4	120	0.88 (0.85, 0.98)	
501425	7	4	120	0.96 (0.92, 1.00)	
501425	8	2	120	0.89 (0.81, 0.96)	
501425	9	2	120	0.98 (0.96, 0.99)	
501425	10	1	120	0.91 (0.91, 0.91)	
501425	11	1	120	0.93 (0.93, 0.93)	
501425	12	1	120	1.00 (1.00, 1.00)	
501425	13	1	120	0.98 (0.98, 0.98)	
501425	14	3	120	1.00 (0.99, 1.00)	
501425	15	2	120	0.92 (0.88, 0.96)	
501425	16	2	120	0.95 (0.94, 0.96)	
501431	1	38	55	1.00 (0.80, 1.00)	Trace/density plots do not show convergence
501431	2	48	55	1.00 (0.86, 1.00)	
501431	3	15	55	0.99 (0.85, 1.00)	
501431	4	4	55	0.89 (0.75, 1.00)	
501431	5	18	55	0.99 (0.79, 1.00)	
501431	6	5	55	0.99 (0.98, 1.00)	
501431	7	3	55	1.00 (0.99, 1.00)	
501431	8	3	55	0.99 (0.93, 1.00)	
501431	9	1	55	0.96 (0.96, 0.96)	
501431	10	1	55	1.00 (1.00, 1.00)	
501431	11	1	55	1.00 (1.00, 1.00)	

Supplementary Table S5. Characteristics of networks that failed to run using one of the models

Characteristics of the networks that failed to run						Indicator of whether the network/treatment failed to run for each model	
**Network ID**	**Number of treatments**	**Number of studies**	**Number of possible (direct and indirect) comparisons**	**Number of total direct comparisons[a]**	**Number of unique direct comparisons[a]**	**Arm-synthesis model 1**	**Arm-synthesis model 2**
479603	45	83	990	95	60	No	Yes
501217[b]	6	38	15	42	10	Yes	Yes
501430[b]	10	19	45	25	12	Yes	Yes

[a] Number of total direct comparisons counts the number of direct comparisons across all studies, whereas the number of unique direct comparisons does not take into account the number of studies. For example, if a network includes 3 studies; studies A and B compare treatments 1, 2, 3, whereas study C compares treatment 1, 2. There are 3 direct comparisons (i.e., 1vs2, 1vs3 and 2vs3) and 7 total direct comparisons (1vs2: 3 studies, 1vs3: 2 studies, 2vs3: 2 studies).

[b] No output was generated and the following error message was obtained: “Error in jags.model(file = textConnection(modelstring), data = data.jags, : Error in node (a(aAR[1]/(a1-AR[1]))/AR[1]); Invalid parent values”

Supplementary Table S6. Comparison of the difference between the Surface Under the Cumulative RAnking curve values (SUCRA) (%) or P-score (%) between the synthesis models (comparing column model to row model) after selecting the treatment ranked as 1 (i.e. the treatment with the highest SUCRA / p-score value) for the model in the row and retaining the corresponding SUCRA/P-score from the other synthesis models [a].

Rank 1 obtained from model below # networks (# observations)	Contrast-synthesis model 1	Contrast-synthesis model 2	Contrast-synthesis model 3*	Arm-synthesis model 1	Arm-synthesis model 2
Contrast-synthesis model 1	Ref	0.89 (00.51, 01.28)	1.26 (00.66, 01.86)	0.00 (-1.02, 01.03)	-9.59 (-12.50, -6.68)
118 (560)	-	p<0.001	p<0.001	p=0.995	p<0.001
Contrast-synthesis model 2	-0.89 (-1.28, -0.51)	Ref	0.37 (-0.18, 00.91)	-0.89 (-1.88, 00.10)	-10.48 (-13.38, -7.58)
118 (560)	p<0.001	-	p=0.188	p=0.080	p<0.001
Contrast-synthesis model 3	-1.88 (-2.40, -1.36)	-0.98 (-1.48, -0.49)	Ref	-1.63 (-2.76, -0.51)	-11.48 (-14.38, -8.57)
118 (560)	p<0.001	p<0.001	-	p=0.005	p<0.001
Arm-synthesis model 1	-2.43 (-3.59, -1.27)	-1.74 (-2.89, -0.60)	-1.04 (-2.28, 00.19)	Ref	-11.79 (-14.83, -8.75)
116 (554)	p<0.001	p=0.003	p=0.098	-	p<0.001
Arm-synthesis model 2	-3.95 (-7.72, -0.17)	-3.42 (-7.17, 00.34)	-3.01 (-6.80, 00.77)	-3.35 (-7.26, 00.57)	Ref
90 (450)	p=0.040	p=0.075	p=0.119	p=0.094	-

[a] For example, if we keep the treatments with the highest SUCRA value (i.e. treatment ranked as 1) using arm-synthesis model 2 (last row) and compare the SUCRA values to contrast-synthesis model 1 (2nd column), we see that, on average, the SUCRA values obtained from contrast-synthesis model 1 are 3.95 units lower (95% CI: -7.72, -0.17) than those obtained from arm-synthesis model 2.

[b] Note that this corresponds to the p-score for contrast-synthesis model 3.

Factors that modify the ratio of the odds ratio between the models

We found that the association between the synthesis models and the odds ratio was modified by the 'ratio of the number of treatments to the number of studies' (Supplementary Table S7a), by the 'ratio of the number of studies to the number of unique direct comparisons' (Supplementary Tables S7c), and by the 'proportion of arms in the network with fewer than 10 events' (Supplementary Table S7d). For the ratio of the number of treatments to the number of studies, and the proportion of arms in the network with fewer than 10 events, compared to CSM1, the odds ratio was similar for CSM2, but showed less benefit for the other synthesis models. On the other hand, for the ratio of the number of studies to the number of unique direct comparisons, compared to CSM1, again the odds ratio was similar for CSM 2, but showed more benefit for the other synthesis models. The 'ratio of the number of treatments to the number of unique direct comparisons' (Supplementary Table S7b) did not appear to modify the association between the synthesis models and the odds ratios.

Supplementary Table S7. Results to assess whether the following factors modified the ratio of the odds ratio between the models.

Supplementary Table S7a. Ratio of the number of treatments to the number of studies

	Number of networks	Number of treatment comparisons	Ratio of the OR [a]	(95% CI)	Comparison of the ratio of the OR against CSM1[b]	(95% CI)
**Contrast-synthesis model 1**	118	867	2.07	(1.31 to 3.26)	Ref	
**Contrast-synthesis model 2**	118	867	2.10	(1.31 to 3.38)	1.02	(0.60 to 1.72)
**Contrast-synthesis model 3**	118	867	1.19	(0.91 to 1.56)	0.59	(0.41 to 0.84)
**Arm-synthesis model 1**	116	853	1.15	(0.77 to 1.71)	0.56	(0.36 to 0.90)
**Arm-synthesis model 2**	90	557	1.02	(0.78 to 1.34)	0.51	(0.35 to 0.73)

Abbreviations: CI, Confidence Interval; CSM1, contrast-synthesis model 1; OR, odds ratio.
For the ratio of the number of treatments to the number of studies, the value of the 25th percentile is 0.24, the value of the 75th percentile is 0.54 and the difference (75th percentile – 25th percentile) is 0.30.
[a] Ratio of the odds ratios for each model when the value of the effect modifier is set to the 25th and 75th percentiles.

[b] Ratio of the odds ratios comparing each model to the reference model for a shift in the effect modifier from the 25th to the 75th percentile.

Supplementary Table S7b. Ratio of the number of treatments to the number of unique direct comparisons

	Number of networks	Number of treatment comparisons	Ratio of the OR [a]	(95% CI)	Comparison of the ratio of the OR against CSM1[b]	(95% CI)
**Contrast-synthesis model 1**	118	867	1.27	(0.80 to 2.01)	Ref	
**Contrast-synthesis model 2**	118	867	1.26	(0.78 to 2.04)	1.00	(0.58 to 1.73)
**Contrast-synthesis model 3**	118	867	1.11	(0.85 to 1.45)	0.87	(0.60 to 1.28)
**Arm-synthesis model 1**	116	853	1.11	(0.74 to 1.66)	0.88	(0.54 to 1.43)
**Arm-synthesis model 2**	90	557	1.06	(0.81 to 1.39)	0.84	(0.57 to 1.23)

Abbreviations: CI, Confidence Interval; CSM1, contrast-synthesis model 1; OR, odds ratio.
For the ratio of the number of treatments to the number of unique direct comparisons, the value of the 25th percentile is 0.60, the value of the 75th percentile is 1.00 and the difference (75th percentile – 25th percentile) is 0.40.

[a] Ratio of the odds ratios for each model when the value of the effect modifier is set to the 25th and 75th percentiles.
[b] Ratio of the odds ratios comparing each model to the reference model for a shift in the effect modifier from the 25th to the 75th percentile.

Supplementary Table S7c. Ratio of the number of studies to the number of unique direct comparisons

	Number of networks	Number of treatment comparisons	Ratio of the OR [a]	(95% CI)	Comparison of the ratio of the OR against CSM1[b]	(95% CI)
**Contrast-synthesis model 1**	118	867	0.81	(0.68 to 0.97)	Ref	
**Contrast-synthesis model 2**	118	867	0.80	(0.67 to 0.97)	0.99	(0.79 to 1.25)
**Contrast-synthesis model 3**	118	867	0.96	(0.87 to 1.05)	1.18	(1.01 to 1.39)
**Arm-synthesis model 1**	116	853	0.97	(0.83 to 1.13)	1.20	(0.98 to 1.47)
**Arm-synthesis model 2**	90	557	0.99	(0.90 to 1.08)	1.23	(1.05 to 1.44)

Abbreviations: CI, Confidence Interval; CSM1, contrast-synthesis model 1; OR, odds ratio.
For the ratio of the number of studies to the number of unique direct comparisons, the value of the 25th percentile is 1.38, the value of the 75th percentile is 2.79 and the difference (75th percentile – 25th percentile) is 1.41.

[a] Ratio of the odds ratios for each model when the value of the effect modifier is set to the 25th and 75th percentiles.

[b] Ratio of the odds ratios comparing each model to the reference model for a shift in the effect modifier from the 25th to the 75th percentile.

Supplementary Table S7d. Proportion of arms in the network with fewer than 10 events

	Number of networks	Number of treatment comparisons	Ratio of the OR [a]	(95% CI)	Comparison of the ratio of the OR against CSM1[b]	(95% CI)
**Contrast-synthesis model 1**	118	867	2.44	(1.51 to 3.94)	Ref	
**Contrast-synthesis model 2**	118	867	2.49	(1.52 to 4.10)	1.02	(0.58 to 1.81)
**Contrast-synthesis model 3**	118	867	1.35	(1.02 to 1.79)	0.55	(0.37 to 0.82)
**Arm-synthesis model 1**	116	853	0.97	(0.64 to 1.47)	0.39	(0.24 to 0.65)
**Arm-synthesis model 2**	90	557	1.60	(1.20 to 2.13)	0.65	(0.44 to 0.97)

Abbreviations: CI, Confidence Interval; CSM1, contrast-synthesis model 1; OR, odds ratio.
For the proportion of arms in the network with fewer than 10 events, the value of the 25th percentile is 0.00, the value of the 75th percentile is 0.25 and the difference (75th percentile – 25th percentile) is 0.25.

[a] Ratio of the odds ratios for each model when the value of the effect modifier is set to the 25th and 75th percentiles.

[b] Ratio of the odds ratios comparing each model to the reference model for a shift in the effect modifier from the 25th to the 75th percentile.

Factors that modify the ratio of the standard errors between the models

We found that all specified factors modified the associations between the synthesis models and the estimates of the standard errors (Supplementary Table S8). For the 'ratio of the number of treatments to the number of studies' (Table S8a) and 'the ratio of the number of treatments to the number of unique direct comparisons' (Table S8b), compared to CSM1, the magnitudes of the standard errors were smaller for CSM2, CSM3 and ASM1 but slightly larger for ASM2. For the 'ratio of the number of studies to the number of unique direct comparisons' (Table S8c), compared to CSM1, the magnitude of the standard errors was slightly smaller for ASM2 but were slightly larger for CSM2, CSM3 and ASM1. For the 'proportion of arms in the network with fewer than 10 events' (Table S8d), the magnitude of the standard error was similar for CSM2 and CSM3 but substantially smaller for ASM1 and ASM2.

Supplementary Table S8. Results to assess whether the factors modified the ratios of the SE(log(OR)) between the models.

Supplementary Table S8a. Ratio of the number of treatments to the number of studies

	Number of networks	Number of treatment comparisons	Ratio of the SE(log(OR)) [a]	(95% CI)	Comparison of the ratio of the SE(log(OR)) against CSM1 [b]	(95% CI)
**Contrast-synthesis model 1**	118	867	1.88	(1.55 to 2.29)	Ref	
**Contrast-synthesis model 2**	118	867	1.62	(1.33 to 1.96)	0.86	(0.85 to 0.88)
**Contrast-synthesis model 3**	118	867	1.53	(1.25 to 1.86)	0.82	(0.78 to 0.85)
**Arm-synthesis model 1**	116	853	1.61	(1.32 to 1.96)	0.86	(0.82 to 0.89)
**Arm-synthesis model 2**	90	557	2.11	(1.73 to 2.59)	1.12	(1.06 to 1.18)

Abbreviations: CI, Confidence Interval; CSM1, contrast-synthesis model 1; OR, odds ratio; SE, Standard Error.

For Ratio of the number of treatments to the number of studies, the value of the 25th percentile is 0.24, the value of the 75th percentile is 0.54 and the difference (75th percentile – 25th percentile) is 0.30.

[a] Ratio of the SE(log(OR)) for each model when the value of the effect modifier is set to the 25th and 75th percentiles.

[b] Ratio of the SE(log(OR)) comparing each model to the reference model for a shift in the effect modifier from the 25th to the 75th percentile.

Supplementary Table S8b. Ratio of the number of treatments to the number of unique direct comparisons

	Number of networks	Number of treatment comparisons	Ratio of the SE(log(OR)) [a]	(95% CI)	Comparison of the ratio of the SE(log(OR)) against CSM1 [b]	(95% CI)
**Contrast-synthesis model 1**	118	867	1.31	(1.06 to 1.62)	Ref	
**Contrast-synthesis model 2**	118	867	1.15	(0.93 to 1.42)	0.87	(0.86 to 0.89)
**Contrast-synthesis model 3**	118	867	1.10	(0.88 to 1.36)	0.83	(0.79 to 0.87)
**Arm-synthesis model 1**	116	853	1.25	(1.01 to 1.55)	0.95	(0.91 to 0.99)
**Arm-synthesis model 2**	90	557	1.36	(1.10 to 1.69)	1.04	(0.98 to 1.10)

Abbreviations: CI, Confidence Interval; CSM1, contrast-synthesis model 1; OR, odds ratio; SE, Standard Error.

For Ratio of the number of treatments to the number of unique direct comparisons, the value of the 25th percentile is 0.60, the value of the 75th percentile is 1.00 and the difference (75th percentile – 25th percentile) is 0.40.

[a] Ratio of the SE(log(OR)) for each model when the value of the effect modifier is set to the 25th and 75th percentiles.

[b] Ratio of the SE(log(OR)) comparing each model to the reference model for a shift in the effect modifier from the 25th to the 75th percentile.

Supplementary Table S8c. Ratio of the number of studies to the number of unique direct comparisons

	Number of networks	Number of treatment comparisons	Ratio of the SE(log(OR)) [a]	(95% CI)	Comparison of the ratio of the SE(log(OR)) against CSM1 [b]	(95% CI)
**Contrast-synthesis model 1**	118	867	0.86	(0.80 to 0.92)	Ref	
**Contrast-synthesis model 2**	118	867	0.87	(0.82 to 0.94)	1.02	(1.01 to 1.03)
**Contrast-synthesis model 3**	118	867	0.89	(0.83 to 0.95)	1.04	(1.02 to 1.06)
**Arm-synthesis model 1**	116	853	0.89	(0.83 to 0.96)	1.05	(1.03 to 1.07)
**Arm-synthesis model 2**	90	557	0.83	(0.77 to 0.89)	0.97	(0.95 to 0.99)

Abbreviations: CI, Confidence Interval; CSM1, contrast-synthesis model 1; OR, odds ratio; SE, Standard Error.
For Ratio of the number of studies to the number of unique direct comparisons, the value of the 25th percentile is 1.38, the value of the 75th percentile is 2.79 and the difference (75th percentile – 25th percentile) is 1.41.

[a] Ratio of the SE(log(OR)) for each model when the value of the effect modifier is set to the 25th and 75th percentiles.

[b] Ratio of the SE(log(OR)) comparing each model to the reference model for a shift in the effect modifier from the 25th to the 75th percentile.

Supplementary Table S8d. Proportion of arms in the network with fewer than 10 events

	Number of networks	Number of treatment comparisons	Ratio of the SE(log(OR)) [a]	(95% CI)	Comparison of the ratio of the SE(log(OR)) against CSM1 [b]	(95% CI)
**Contrast-synthesis model 1**	118	867	1.33	(1.06 to 1.67)	Ref	
**Contrast-synthesis model 2**	118	867	1.32	(1.05 to 1.66)	0.99	(0.97 to 1.02)
**Contrast-synthesis model 3**	118	867	1.30	(1.03 to 1.63)	0.97	(0.93 to 1.02)
**Arm-synthesis model 1**	116	853	1.05	(0.83 to 1.31)	0.78	(0.75 to 0.82)
**Arm-synthesis model 2**	90	557	1.05	(0.83 to 1.32)	0.78	(0.73 to 0.84)

Abbreviations: CI, Confidence Interval; CSM1, contrast-synthesis model 1; OR, odds ratio; SE, Standard Error.
For Proportion of arms in the network with fewer than 10 events, the value of the 25th percentile is 0.00, the value of the 75th percentile is 0.25 and the difference (75th percentile – 25th percentile) is 0.25.

[a] Ratio of the SE(log(OR)) for each model when the value of the effect modifier is set to the 25th and 75th percentiles.

[b] Ratio of the SE(log(OR)) comparing each model to the reference model for a shift in the effect modifier from the 25th to the 75th percentile.

Factors that modify the ratio of the SUCRA values and treatment ranks between the models

We found that all factors modified the association between the synthesis models and the magnitude of the SUCRA (%) (Supplementary Tables S9) and treatment rank (Supplementary Tables S10). For all factors, the difference in the SUCRA values and treatment ranks for CSM2 and CSM3 and ASM1 were similar to CSM1, whereas for ASM2 we observed large differences.

Supplementary Table S9. Results to assess whether the following factors modified the differences in the SUCRA values between the models.

Supplementary Table S9a. Ratio of the number of treatments to the number of studies

	Number of networks	Number of treatment comparisons	Difference in SUCRA values (%) [a]	(95% CI)	Comparison of the difference in the SUCRA values (%) against CSM1 [b]	(95% CI)
**Contrast-synthesis model 1**	118	118	-2.43	(-4.80 to -0.06)	Ref	
**Contrast-synthesis model 2**	118	118	-1.10	(-3.44 to 1.25)	1.30	(0.80 to 1.80)
**Contrast-synthesis model 3**	118	118	-1.37	(-3.82 to 1.08)	1.03	(0.18 to 1.89)
**Arm-synthesis model 1**	116	116	-2.17	(-4.90 to 0.57)	0.25	(-1.21 to 1.71)
**Arm-synthesis model 2**	90	90	-9.64	(-14.02 to -5.25)	-7.03	(-10.67 to -3.38)

Abbreviations: CI, Confidence Interval; CSM1, contrast-synthesis model 1; SUCRA, surface under the cumulative ranking curve.
For the ratio of the number of treatments to the number of studies, the value of the 25th percentile is 0.22, the value of the 75th percentile is 0.53 and the difference (75th percentile – 25th percentile) is 0.31.
[a] Difference in SUCRA values for each model when the value of the factor is set to the 25th and 75th percentiles.
[b] Difference in the SUCRA values comparing each model to the reference model for a shift in the factor from the 25th to the 75th percentile.

Supplementary Table S9b. Ratio of the number of treatments to the number of unique direct comparisons

	Number of networks	Number of treatment comparisons	Difference in SUCRA values (%)[a]	(95% CI)	Comparison of the difference in the SUCRA values (%) against CSM1 [b]	(95% CI)
**Contrast-synthesis model 1**	118	118	-0.80	(-3.62 to 2.01)	Ref	
**Contrast-synthesis model 2**	118	118	0.28	(-2.50 to 3.06)	1.11	(0.45 to 1.76)
**Contrast-synthesis model 3**	118	118	0.18	(-2.72 to 3.08)	1.00	(-0.07 to 2.07)
**Arm-synthesis model 1**	116	116	-1.66	(-4.88 to 1.56)	-0.89	(-2.68 to 0.91)
**Arm-synthesis model 2**	90	90	-6.29	(-11.74 to -0.84)	-5.63	(-10.48 to -0.78)

Abbreviations: CI, Confidence Interval; CSM1, contrast-synthesis model 1.
For the ratio of the number of treatments to the number of unique direct comparisons, the value of the 25th percentile is 0.64, the value of the 75th percentile is 1.12, and the difference (75th percentile – 25th percentile) is 0.48.
[a] SUCRA values for each model when the value of the factor is set to the 25th and 75th percentiles.
[b] Difference in the SUCRA values comparing each model to the reference model for a shift in the factor from the 25th to the 75th percentile.

Supplementary Table S9c. Ratio of the number of studies to the number of unique direct comparisons

	Number of networks	Number of treatment comparisons	Difference in SUCRA values (%)[a]	(95% CI)	Comparison of the difference in the SUCRA values (%) against CSM1 [b]	(95% CI)
**Contrast-synthesis model 1**	118	118	0.69	(-0.29 to 1.67)	Ref	
**Contrast-synthesis model 2**	118	118	0.48	(-0.48 to 1.45)	-0.20	(-0.43 to 0.02)
**Contrast-synthesis model 3**	118	118	0.53	(-0.47 to 1.54)	-0.16	(-0.52 to 0.21)
**Arm-synthesis model 1**	116	116	0.62	(-0.50 to 1.74)	-0.07	(-0.68 to 0.55)
**Arm-synthesis model 2**	90	90	2.22	(0.40 to 4.04)	1.53	(-0.03 to 3.09)

Abbreviations: CI, Confidence Interval; CSM1, contrast-synthesis model 1.
For the ratio of the number of studies to the number of unique direct comparisons, the value of the 25th percentile is 1.62, the value of the 75th percentile is 3.29 and the difference (75th percentile -25th percentile) is 1.67.
[a] SUCRA values for each model when the value of the factor is set to the 25th and 75th percentiles.

[b] Difference in the SUCRA values comparing each model to the reference model for a shift in the factor from the 25th to the 75th percentile.

Supplementary Table S9d. Proportion of arms in the network with fewer than 10 events

	Number of networks	Number of treatment comparisons	Difference in SUCRA values (%)[a]	(95% CI)	Comparison of the difference in the SUCRA values (%) against CSM1 [b]	(95% CI)
**Contrast-synthesis model 1**	118	118	1.78	(-0.45 to 4.01)	Ref	
**Contrast-synthesis model 2**	118	118	1.93	(-0.27 to 4.13)	0.15	(-0.38 to 0.68)
**Contrast-synthesis model 3**	118	118	1.55	(-0.75 to 3.84)	-0.23	(-1.06 to 0.60)
**Arm-synthesis model 1**	116	116	2.16	(-0.41 to 4.72)	0.38	(-1.04 to 1.79)
**Arm-synthesis model 2**	90	90	6.91	(2.53 to 11.29)	5.13	(1.30 to 8.96)

Abbreviations: CI, Confidence Interval; CSM1, contrast-synthesis model 1.
For the proportion of arms in the network with fewer than 10 events, the value of the 25th percentile is 0.00, the value of the 75th percentile is 0.22 and the difference (75th percentile – 25th percentile) is 0.22.
[a] SUCRA values for each model when the value of the factor is set to the 25th and 75th percentiles.

[b] Difference in the SUCRA values comparing each model to the reference model for a shift in the factor from the 25th to the 75th percentile.

Supplementary Table S10. Results to assess whether the following factors modified the differences in the ranks between the models.

Supplementary Table S10a. Ratio of the number of treatments to the number of studies

	Number of networks	Number of treatment comparisons	Difference in the Ranks [a]	(95% CI)	Comparison of the difference in the ranks against CSM1 [b]	(95% CI)
**Contrast-synthesis model 1**	118	118	0.00	(-0.13 to 0.13)	Ref	
**Contrast-synthesis model 2**	118	118	0.00	(-0.13 to 0.13)	0.00	(-0.17 to 0.17)
**Contrast-synthesis model 3**	118	118	-0.05	(-0.17 to 0.08)	-0.04	(-0.21 to 0.13)
**Arm-synthesis model 1**	116	116	0.04	(-0.09 to 0.17)	0.04	(-0.13 to 0.21)
**Arm-synthesis model 2**	90	90	0.36	(0.22 to 0.49)	0.35	(0.17 to 0.53)

Abbreviations: CI, Confidence Interval; CSM1, contrast-synthesis model 1; $\boldsymbol{\tau}$, square-root of the between-study heterogeneity.
For the ratio of the number of treatments to the number of studies, the value of the 25th percentile is 0.22 and the value of the 75th percentile is 0.53 and the difference (75th percentile – 25th percentile) is 0.31.
[a] Rank for each model when the value of the factor is set to the 25th and 75th percentiles.
[b] Difference in the ranks comparing each model to the reference model for a shift in the factor from the 25th to the 75th percentile.

Supplementary Table S10b. Ratio of the number of treatments to the number of unique direct comparisons

	Number of networks	Number of treatment comparisons	Difference in the Ranks [a]	(95% CI)	Comparison of the difference in the ranks against CSM1 [b]	(95% CI)
**Contrast-synthesis model 1**	118	118	0.00	(-0.15 to 0.15)	Ref	
**Contrast-synthesis model 2**	118	118	0.00	(-0.15 to 0.15)	0.00	(-0.22 to 0.22)
**Contrast-synthesis model 3**	118	118	-0.05	(-0.20 to 0.10)	-0.05	(-0.26 to 0.17)
**Arm-synthesis model 1**	116	116	-0.02	(-0.17 to 0.13)	-0.02	(-0.24 to 0.19)
**Arm-synthesis model 2**	90	90	0.11	(-0.06 to 0.27)	0.11	(-0.12 to 0.34)

Abbreviations: CI, Confidence Interval; CSM1, contrast-synthesis model 1; $\boldsymbol{\tau}$, square-root of the between-study heterogeneity.
For the ratio of the number of treatments to the number of unique direct comparisons, the value of the 25th percentile is 0.64, the value of the 75th percentile is 1.12 and the difference (75th percentile – 25th percentile) is 0.48.
[a] Rank for each model when the value of the factor is set to the 25th and 75th percentiles.
[b] Difference in the ranks comparing each model to the reference model for a shift in the factor from the 25th to the 75th percentile.

Supplementary Table S10c. Ratio of the number of studies to the number of unique direct comparisons

	Number of networks	Number of treatment comparisons	Difference in the Ranks [a]	(95% CI)		Comparison of the difference in the ranks against CSM1 [b]	(95% CI)	
**Contrast-synthesis model 1**	118	118	0.00	(-0.05 to	0.05)	Ref		
**Contrast-synthesis model 2**	118	118	0.00	(-0.05 to	0.05)	0.00	(-0.07 to	0.07)
**Contrast-synthesis model 3**	118	118	0.01	(-0.04 to	0.07)	0.01	(-0.06 to	0.09)
**Arm-synthesis model 1**	116	116	-0.03	(-0.09 to	0.02)	-0.03	(-0.11 to	0.04)
**Arm-synthesis model 2**	90	90	-0.12	(-0.17 to	-0.07)	-0.12	(-0.19 to	-0.05)

Abbreviations: CI, Confidence Interval; CSM1, contrast-synthesis model 1; τ, square-root of the between-study heterogeneity.
For the ratio of the number of studies to the number of unique direct comparisons, the value of the 25th percentile is 1.62, the value of the 75th percentile is 3.29 and the difference (75th percentile – 25th percentile) is 1.67.
[a] Rank for each model when the value of the factor is set to the 25th and 75th percentiles.
[b] Difference in the ranks comparing each model to the reference model for a shift in the factor from the 25th to the 75th percentile.

Supplementary Table S10d. Proportion of arms in the network with fewer than 10 events

	Number of networks	Number of treatment comparisons	Difference in the Ranks [a]	(95% CI)		Comparison of the difference in the ranks against CSM1 [b]	(95% CI)	
**Contrast-synthesis model 1**	118	118	0.00	(-0.12 to	0.12)	Ref		
**Contrast-synthesis model 2**	118	118	0.00	(-0.12 to	0.12)	0.00	(-0.17 to	0.17)
**Contrast-synthesis model 3**	118	118	-0.02	(-0.14 to	0.10)	-0.02	(-0.19 to	0.15)
**Arm-synthesis model 1**	116	116	0.00	(-0.12 to	0.12)	0.00	(-0.17 to	0.17)
**Arm-synthesis model 2**	90	90	-0.17	(-0.31 to	-0.04)	-0.17	(-0.35 to	0.00)

Abbreviations: CI, Confidence Interval; CSM1, contrast-synthesis model 1; τ, square-root of the between-study heterogeneity.
For the proportion of arms in the networks with fewer than 10 events, the value of the 25th percentile is 0.00, the value of the 75th percentile is 0.22 and the difference (75th percentile – 25th percentile) is 0.22.
[a] Rank for each model when the value of the factor is set to the 25th and 75th percentiles.
[b] Difference in the ranks comparing each model to the reference model for a shift in the factor from the 25th to the 75th percentile.

Factors that modify the ratio of the between-study heterogeneity between the models

The relationship between the magnitude of the between-study heterogeneity standard-deviation and the synthesis models was not modified by any of the four factors (Supplementary Tables S11).

Supplementary Table S11. Results to assess whether the following factors modified the differences in the square-root of the between study heterogeneity (?) between the models

Supplementary Table S11a. Ratio of the number of treatments to the number of studies

	Number of networks	Number of treatments	Ratio of τ [a]	(95% CI)	Comparison of the ratio of τ against CSM1 [b]	(95% CI)
**Contrast-synthesis model 1**	118	118	1.36	(0.97 to 1.91)	Ref	
**Contrast-synthesis model 2**	118	118	1.05	(0.75 to 1.48)	0.77	(0.48 to 1.25)
**Contrast-synthesis model 3**	118	118	0.84	(0.60 to 1.18)	0.62	(0.38 to 1.00)

Abbreviations: CI, Confidence Interval; CSM1, contrast-synthesis model 1; τ, square-root of the between-study heterogeneity.
For the ratio of the number of treatments to the number of studies, the value of the 25th percentile is 0.22 and the value of the 75th percentile is 0.53 and the difference (75th percentile – 25th percentile) is 0.31.
[a] Estimate of the square-root of the between-study heterogeneity for each model when the value of the factor is set to the 25th and 75th percentiles.
[b] Ratio of the square-root of the between-study heterogeneity when the factor is set to its value at the 75th percentile and comparing each model to contrast-synthesis model 1 after removing out any systematic differences between the models at the 25th percentile for that factor.

Supplementary Table S11b. Ratio of the number of treatments to the number of unique direct comparisons

	Number of networks	Number of treatments	Ratio of τ [a]	(95% CI)	Comparison of the ratio of τ against CSM1 [b]	(95% CI)
**Contrast-synthesis model 1**	118	118	1.36	(0.90 to 2.07)	Ref	
**Contrast-synthesis model 2**	118	118	1.08	(0.71 to 1.65)	0.80	(0.44 to 1.44)
**Contrast-synthesis model 3**	118	118	0.67	(0.44 to 1.02)	0.49	(0.27 to 0.89)

Abbreviations: CI, Confidence Interval; CSM1, contrast-synthesis model 1; τ, square-root of the between-study heterogeneity.
For the ratio of the number of treatments to the number of unique direct comparisons, the value of the 25th percentile is 0.64, the value of the 75th percentile is 1.12 and the difference (75th percentile – 25th percentile) is 0.48.
[a] Estimate of the square-root of the between-study heterogeneity for each model when the value of the factor is set to the 25th and 75th percentiles.
[b] Ratio of the square-root of the between-study heterogeneity when the factor is set to its value at the 75th percentile and comparing each model to the contrast-synthesis model 1 after removing out any systematic differences between the models at the 25th percentile for that factor.

Supplementary Table S11c. Ratio of the number of studies to the number of unique direct comparisons

	Number of networks	Number of treatments	Ratio of τ [a]	(95% CI)	Comparison of the ratio of τ against CSM1 [b]	(95% CI)
**Contrast-synthesis model 1**	118	118	0.96	(0.83 to 1.11)	Ref	
**Contrast-synthesis model 2**	118	118	1.01	(0.87 to 1.16)	1.04	(0.85 to 1.28)
**Contrast-synthesis model 3**	118	118	1.02	(0.88 to 1.18)	1.06	(0.87 to 1.30)

Abbreviations: CI, Confidence Interval; CSM1, contrast-synthesis model 1; τ, square-root of the between-study heterogeneity.
For the ratio of the number of studies to the number of unique direct comparisons, the value of the 25th percentile is 1.62, the value of the 75th percentile is 3.29 and the difference (75th percentile – 25th percentile) is 1.67.
[a] Estimate of the square-root of the between-study heterogeneity for each model when the value of the factor is set to the 25th and 75th percentiles.
[b] Ratio of the square-root of the between-study heterogeneity when the factor is set to its value at the 75th percentile and comparing each model to the contrast-synthesis model 1 after removing out any systematic differences between the models at the 25th percentile for that factor.

Supplementary Table S11d. Proportion of arms in the network with fewer than 10 events

	Number of networks	Number of treatments	Ratio of τ [a]	(95% CI)	Comparison of the ratio of τ against CSM1 [b]	(95% CI)
**Contrast-synthesis model 1**	118	118	1.33	(0.96 to 1.84)	Ref	
**Contrast-synthesis model 2**	118	118	1.31	(0.95 to 1.81)	0.98	(0.62 to 1.55)
**Contrast-synthesis model 3**	118	118	1.70	(1.23 to 2.35)	1.28	(0.81 to 2.02)

Abbreviations: CI, Confidence Interval; CSM1, contrast-synthesis model 1; τ, square-root of the between-study heterogeneity.
For the proportion of arms in the network with fewer than 10 events, the value of the 25th percentile is 0.00, the value of the 75th percentile is 0.22 and the difference (75th percentile – 25th percentile) is 0.22.
[a] Ratio of the square-root of the between-study heterogeneity for each model when the value of the factor is set to the 25th and 75th percentiles.
[b] Ratio of the square-root of the between-study heterogeneity when the factor is set to its value at the 75th percentile and comparing each model to the contrast-synthesis model 1 after removing out any systematic differences between the models at the 25th percentile for that factor.

Supplementary Table S12. Data for the NMA empirical paper.

See Excel file

R code to fit the models to one of the eligible datasets.

Note that the dataset is available for downloading as a Stata datafile.

```
#############################################################################
# Runs the NMA models on a single dataset
#############################################################################
#remove all objects in R
rm(list=ls())

# Load required R packages
#packages to read Stata dataset
install.packages("readstata13")
library(readstata13)
install.packages("foreign")
library(foreign)

# gemtc for contrast based analysis
install.packages("gemtc")
library("gemtc")

# netmeta for frequentist analysis
install.packages("netmeta")
library("netmeta")

# rjags for analysis
install.packages("rjags")
library("rjags")

# pcnetmeta for arm based analysis
install.packages("pcnetmeta")
library("pcnetmeta")

# lattice for graphs
install.packages("lattice")
library(lattice)
#
#############################################################################
# Paths and data
# set project (NMA) directory
NMA_dir <- "."
# set writing (R) directory
writing_dir <- "From_R"
# set reading (Stata) directory
read_dir <- "."

# read data
mydata <- read.dta13(file.path(NMA_dir, read_dir, " Supplementary File Data
479661.dta"))

myvars <- c("PMID", "study", "id", "n", "r", "tx", "txName",
"Turner_Intervention", "Turner_Outcome", "reference_tx")

metadata <- mydata[myvars]

# Fix up treatment names
# no spaces
```

```
metadata$treatment <- gsub(" ", "_", metadata$txName)
# no dashes
metadata$treatment <- gsub("-", "_", metadata$txName)
# no pluses
metadata$treatment <- gsub("[[:punct:]]", "_", metadata$txName)
# try getting rid of non-alphanumeric
metadata$treatment <- gsub("[^[:alnum:]]", "_", metadata$txName)
# still some oddities
metadata$treatment <- gsub("[^a-zA-Z0-9]", "_", metadata$txName)

names(metadata)[names(metadata)=="tx"] <- "treatment"
names(metadata)[names(metadata)=="r"] <- "responders"
names(metadata)[names(metadata)=="n"] <- "sampleSize"

# convert sampleSize and responders to numeric
metadata <- transform(metadata, treatment=as.integer(treatment))
metadata <- transform(metadata, sampleSize=as.integer(sampleSize))
metadata <- transform(metadata, responders=as.integer(responders))

reference_tx <- 1
metadata.current.PMID <- subset(metadata, metadata$PMID == 479661)

gemtc_network_names <- mtc.network(metadata.current.PMID)

alltreatments <- sort(unique(metadata.current.PMID[,"treatment"]))
gemtc_network_numbers <- mtc.network(metadata.current.PMID)
outcome.type = "mortality"
comparison.type = "pharma-pharma"

############################################################################
# Method: gemtc
# contrast based methods
# non-informative priors set up
gemtc_name <- "gemtc_non_info"
# set up parameters for the non-informative priors and for the model running.
mtcmodel_type <- "consistency"
mtcmodel_factor <- 2.5
mtcmodel_n.chain <- 3
mtcmodel_likelihood <- "binom"
mtcmodel_link <- "logit"
mtcmodel_linearModel <- "random"
mtcmodel_mtc.hy.prior <- mtc.hy.prior("std.dev","dunif",0,10)

mtcresults_n.adapt <- 10000
mtcresults_n.iter <- 3000
mtcresults_thin <- 500
file_prefix <- "gemtc_non_inf"

mtcmodel <- mtc.model(network=gemtc_network_numbers,
                      type=mtcmodel_type,
                      factor=mtcmodel_factor,
                      n.chain=mtcmodel_n.chain,
                      likelihood=mtcmodel_likelihood,
                      link=mtcmodel_link,
                      linearModel=mtcmodel_linearModel,
                      hy.prior=mtcmodel_mtc.hy.prior
)
```

```
mtcresults <- mtc.run(mtcmodel, n.adapt=mtcresults_n.adapt,
n.iter=mtcresults_n.iter, thin=mtcresults_thin)
gelman_diag <- gelman.diag(mtcresults)
gelman_psrf <- as.data.frame(gelman_diag$psrf)
source(file.path(NMA_dir, "R_output.r"))

#Note that the code for file "R_output.r" is provide on page 46 below.

#Turner informative priors set up
#choose defaults
mtcmodel_mtc.hy.prior <- mtc.hy.empirical.lor(outcome.type = outcome.type,
comparison.type = comparison.type)

mtcmodel <- mtc.model(network=gemtc_network_numbers,
                      type=mtcmodel_type,
                      factor=mtcmodel_factor,
                      n.chain=mtcmodel_n.chain,
                      likelihood=mtcmodel_likelihood,
                      link=mtcmodel_link,
                      linearModel=mtcmodel_linearModel,
                      hy.prior=mtcmodel_mtc.hy.prior
)

mtcresults <- mtc.run(mtcmodel, n.adapt=mtcresults_n.adapt,
n.iter=mtcresults_n.iter, thin=mtcresults_thin)
gelman_diag <- gelman.diag(mtcresults)
gelman_psrf <- as.data.frame(gelman_diag$psrf)
source(file.path(NMA_dir, "R_output.r"))

##########################################################################
#Method: netmeta
#frequentist based methods
file_wide.data <- pairwise(treatment,
                          event = responders,
                          n = sampleSize,
                          studlab = study,
                          data = metadata.current.PMID,
                          allstudies = TRUE,
                          sm = "OR")
net.results <- netmeta(TE, seTE, treat1, treat2, studlab,
data=file_wide.data, sm = "OR", reference.group = reference_tx,
all.treatments=TRUE, random=TRUE)
# use netrank to generate pscore
netmeta.pscore <- netrank(net.results)

##########################################################################
# Method: pcnetmeta
## arm based methods
pcnetmeta_model_n.iter <- 3000
pcnetmeta_model_n.thin <- 500
pcnetmeta_model_n.burnin <- 300000
pcnetmeta_model_prior.type <- "unif"
pcnetmeta_model_model <- "hom_eqcor"
pcnetmeta_model_n.adapt <- 1000
```

```
nma.results <- nma.ab.bin(s.id = metadata.current.PMID[,"study"],
                          t.id = metadata.current.PMID[,"treatment"],
                          event.n = metadata.current.PMID[,"responders"],
                          total.n = metadata.current.PMID[,"sampleSize"],
                          higher.better = FALSE,
                          param = c("LOR","rank.prob"),
                          mcmc.samples = TRUE,
                          conv.diag= TRUE,
                          #dic = TRUE,
                          #trace = c("LOR"),
                          postdens = TRUE,
                          n.iter = pcnetmeta_model_n.iter,
                          n.thin = pcnetmeta_model_n.thin,
                          n.burnin = pcnetmeta_model_n.burnin,
                          prior.type = pcnetmeta_model_prior.type,
                          n.adapt = pcnetmeta_model_n.adapt,
                          model = pcnetmeta_model_model)

#setup for pcnetmeta_2
pcnetmeta_model_model <- "het_eqcor"
nma.results <- nma.ab.bin(s.id = metadata.current.PMID[,"study"],
                          t.id = metadata.current.PMID[,"treatment"],
                          event.n = metadata.current.PMID[,"responders"],
                          total.n = metadata.current.PMID[,"sampleSize"],
                          higher.better = FALSE,
                          param = c("LOR","rank.prob"),
                          mcmc.samples = TRUE,
                          conv.diag= TRUE,
                          #dic = TRUE,
                          #trace = c("LOR"),
                          postdens = TRUE,
                          n.iter = pcnetmeta_model_n.iter,
                          n.thin = pcnetmeta_model_n.thin,
                          n.burnin = pcnetmeta_model_n.burnin,
                          prior.type = pcnetmeta_model_prior.type,
                          n.adapt = pcnetmeta_model_n.adapt,
                          model = pcnetmeta_model_model)

##############################################################################
```

```
############################################################################
# R_output.R
# trace plot
trace_plot <- traceplot(mtcresults$samples[, nvar(mtcresults$samples)])

# density plot
for (num.var in colnames(mtcresults$samples[[1]])) {
  xlabel <- ifelse(num.var != "sd.d","logOR","tau")
  density_title <- paste0(479661,"_Density_", num.var)
  density_plot <- densityplot(mtcresults$samples[,num.var])
}
# calculate SUCRA and ranks
myprobs<-rank.probability(mtcresults,preferredDirection = -1)

# convert to data
myprobs.data <- as.data.frame(as.table(myprobs))

# name the headings more nicely
names(myprobs.data)[names(myprobs.data) == "Var1"] <- "treatment"
names(myprobs.data)[names(myprobs.data) == "Var2"] <- "rank"

# name the ranks more nicely
unique.ranks <- unique(myprobs.data$rank)
for(num.ranks in 1:length(unique.ranks)){levels(myprobs.data$rank)[num.ranks]
<- paste("Rank", num.ranks, sep="_")}

# reshape to wide
myprobs.data <- reshape(myprobs.data,
timevar="rank",idvar="treatment",direction="wide")
#  generate cdfs
#  loop over columns
for(num.ranks in 1:length(unique.ranks)){
  num.ranks.plus <- num.ranks + 1
  if (num.ranks == 1) {
    myprobs.data$newcol <- myprobs.data[,num.ranks.plus]
  } else {
    myprobs.data$newcol <- rowSums(myprobs.data[,2:num.ranks.plus])
  }

  # rename column
  names(myprobs.data)[names(myprobs.data) == "newcol"] <-
paste("cdf",num.ranks,sep="_")
}
# generate SUCRA
colstart <- length(unique.ranks) + 2
colend <- (2 * length(unique.ranks))
myprobs.data$SUCRA <- (1/(length(unique.ranks)-
1))*rowSums(myprobs.data[,colstart:colend])

# generate SUCRA ranks
SUCRA.order <- order(-myprobs.data$SUCRA)
myprobs.data <- myprobs.data[SUCRA.order,]
myprobs.data$SUCRA.rank <- 1:nrow(myprobs.data)
myprobs.data$PMID <- 479661
current.SUCRA.brief <- subset(myprobs.data, select=c(PMID, treatment, SUCRA,
SUCRA.rank))
############################################################################
```
